# Comparing the antidiabetic effects and chemical profiles of raw and fermented Chinese Ge-Gen-Qin-Lian decoction by integrating untargeted metabolomics and targeted analysis

**DOI:** 10.1186/s13020-018-0208-7

**Published:** 2018-10-26

**Authors:** Yan Yan, Chenhui Du, Zhenyu Li, Min Zhang, Jin Li, Jinping Jia, Aiping Li, Xuemei Qin, Qiang Song

**Affiliations:** 1 Modern Research Center for Traditional Chinese Medicine of Shanxi University, No. 92, Wucheng Road, Taiyuan, 030006 Shanxi China; 2 School of Traditional Chinese Materia Medica, Shanxi University of Chinese Medicine, No. 121, Daxue Street, Taiyuan, 030619 Shanxi China; 3 College of Chemistry and Chemical Engineering of Shanxi University, No. 92, Wucheng Road, Taiyuan, 030006 Shanxi China

**Keywords:** Ge-Gen-Qin-Lian decoction, Fermentation, Untargeted metabolomics, Targeted analysis, Antidiabetic effects

## Abstract

**Background:**

Microbial fermentation has been widely applied in traditional Chinese medicine (TCM) for thousands of years in China. Various beneficial effects of fermentation for applications in TCM or herbals have been reported, such as enhanced anti-ovarian cancer, antioxidative activity, and neuroprotective effects. Ge-Gen-Qin-Lian decoction (GQD), a classic TCM formula, has been used to treat type 2 diabetes mellitus in China. In this study, GQD was fermented with *Saccharomyces cerevisiae*, and the antidiabetic activities and overall chemical profiles of raw and fermented GQD (FGQD) were systematically compared.

**Methods:**

First, the antidiabetic effects of GQD and FGQD on high-fat diet and streptozotocin (STZ)-induced diabetic rats were compared. Then, high-performance liquid chromatography Q Exactive MS was applied for rapid characterization of the chemical components of GQD. Additionally, we proposed an integrated chromatographic technique based untargeted metabolomics identifying differential chemical markers between GQD and FGQD and targeted analysis determining the fermenting-induced quantitative variation tendencies of chemical marker strategy for overall chemical profiling of raw and fermented GQD.

**Results:**

Both GQD and FGQD displayed effects against HFD and STZ-induced diabetes, and FGQD showed a better recovery trend associated with profound changes in the serum lipoprotein profile and body weight gain. In addition, 133 compounds were characterized from GQD. It was demonstrated that the integrated strategy holistically illuminated 30 chemical markers contributed to the separation of GQD and FGQD, and further elucidated the fermenting-induced chemical transformation mechanisms and inherent chemical connections of secondary metabolites. Although there were no new secondary metabolites in FGQD compared with GQD, the amounts of secondary metabolites, which were mostly deglycosylated, were redistributed in FGQD.

**Conclusion:**

The anti-diabetic activities of GQD could be improved by applying fermentation technology. Moreover, the proposed strategy could serve as a powerful tool for systematically exploring the chemical profiles of raw and fermented formulas.

**Electronic supplementary material:**

The online version of this article (10.1186/s13020-018-0208-7) contains supplementary material, which is available to authorized users.

## Background

Herbal fermentation, which began approximately 4000 years ago in China, is used to produce secondary metabolites from plants in bulk by utilizing the metabolic pathways of microorganisms [1]. Fermented medicinal plants and traditional Chinese medicine (TCM) are attracting increasing attention in East Asia, especially in Taiwan and Korea. During the fermentation of TCM, certain glycosides are deglycosylated into small, hydrophobic molecules that may be more efficacious than the original herbal medicines due to increased absorption and bioavailability of the active components in the body [2–5]. Fermented medicinal plants and traditional herbal medicine have been shown to exhibit enhanced anti-ovarian cancer activity, antioxidative activity, and neuroprotective effects compared to the raw formulas [6–9]. The yeast *Saccharomyces cerevisiae* (SC) is the most widely used organism for fermentation and has been successfully used for the biotransformation of TCM formula [4, 5, 10].

Although various beneficial effects of fermentation applied to TCM or medicinal plants have been reported, systematic comparisons of the pharmacological actions and overall chemical profiles of raw and fermented TCM formulas are scarce. TCM is a complex system comprising hundreds of different compounds. Thus, the most critical difficulty is distinguishing and matching herbal biotransformed secondary metabolites in complex microorganism matrixes. Metabolomics, a novel approach for rapidly identifying the global metabolic composition of biological systems, has been widely used for the overall chemical characterization of herbal medicines [11]. Thus, metabolomics analysis could be used to study the effects of fermentation on TCM. In general, the purpose of untargeted metabolomics is to identify statistically significant differences based on unbiased differential analysis of as many signals as possible [12]. By contrast, targeted quantitative metabolomics is intended mainly to accurately determine metabolites in various samples by comparison with authentic compounds to improve the repeatability, comparability and reproducibility of data [13]. Liquid chromatography coupled with mass spectrometry (LC–MS)-based untargeted metabolomic approach can provide global profiles of abundant (up to hundreds of) secondary metabolites by determining their presence, amount and occasionally their structures [14, 15] and has been successfully used to study the effects of processing on herbal drugs, such as Rehmanniae Radix and Fructus corni [15, 16].

Ge-Gen-Qin-Lian decoction (GQD), a well-known TCM formula, was first recorded in “Treatise on Febrile Diseases” compiled by Zhong-jing Zhang of the Han Dynasty (202 BC-220 AD). GQD consists of four herbs, Pueraria Lobatae Radix, Scutellariae Radix, Coptidis Rhizoma, and Glycyrrhizae Radix et Rhizoma Praeparata cum Melle, in a weight ratio of 8:3:3:2. Extensive chemical studies have shown that flavones (free form and glycosides), flavanones, alkaloids and triterpene saponins are the major compounds in GQD [17, 18]. Modern pharmacological studies have revealed that GQD has antidiabetic effects in vivo and in vitro [19–22]. GQD is also clinically used to treat type 2 diabetes mellitus (T2DM) [23].

Since GQD and SC have a long history and extensive range of use, their safety and efficacy are demonstrated and widely accepted by the public. Here, GQD was fermented using SC, and the antidiabetic effects of GQD and fermented GQD (FGQD) on high-fat diet (HFD) and streptozotocin (STZ)-induced diabetic rats were compared. An integrated strategy based on untargeted and targeted metabolomic analysis was proposed for the overall chemical profiling of raw and fermented GQD. Finally, the correlations of the biological and chemical differences are discussed.

## Methods

### Information on experimental design and resources

The information regarding the experimental design, statistics, and resources used in this study is attached in the minimum standards of reporting checklist (Additional file 1).

### Chemicals, materials and reagents

Acetonitrile (HPLC and MS grade) and methanol (HPLC grade) were purchased from Tedia (Fairfield, USA) and Hanbon (Nanjing, China), respectively. Formic acid (analytical grade) was provided by Aladdin Chemistry Co. Ltd (Shanghai, China). De-ionized water was prepared in-house by a Milli-Q water purification system (Millipore, MA, USA). Other chemicals and reagents were analytical grade. The chemical reference substances (purity > 98%, HPLC–DAD) of 3′-hydroxypuerarin, puerarin, daidzin, daidzein, baicalin, wogonoside, baicalein, wogonin, coptisine, berberine, palmatine, magnoflorine, genistin, genistein, ononin and formononetin were purchased from Chengdu Wei ke-qi Bio-Technology Co., Ltd. (Chengdu, China). Liquiritin, isoliquiritin, liquiritigenin, isoliquiritigenin and glycyrrhizic acid were purchased from Chunqiu Bio-Technology Co., Ltd. (Nanjing, China). Scutellarein (purity > 98%, HPLC–DAD) was isolated, purified and identified in our lab.

Puerariae Lobatae Radix (Gegen), Scutellariae Radix (Huangqin), Coptidis Rhizoma (Huanglian) and Glycyrrhizae Radix et Rhizoma Praepapata Cum Melle (Zhigancao) were purchased from Wan Min pharmacy (Taiyuan, China) and authenticated by Associate Professor Chenhui Du, according to the standard of the Chinese Pharmacopeia (2015 edition). Voucher specimens were deposited in the Modern Research Center for Traditional Chinese Medicine of Shanxi University. SC (CICC 1205) was purchased from the China Center of Industrial Culture Collection (CICC).

### Preparation of GQD and FGQD extracts

Herb pieces of 3200 g (Gegen:Huangqin:Huanglian:Gancao = 8:3:3:2) were immersed in a 10-fold volume of distilled water (w/v) for 0.5 h and then extracted by refluxing two times (40 min, 30 min). For each extract, the decoction was filtered through eight layers of gauze to remove the herbal residue. The two filtrates were combined, condensed under reduced pressure with a rotary evaporator at 70 °C and evaporated to dryness (yield: 28.6%).

Freeze-dried spores of SC were recovered in 25 mL of potato dextrose (PD) medium and then incubated at 28 °C on a rotary shaker at 180×*g* for 24 h. A 20-mL volume of GQD (0.5 g mL^−1^, crude drug per g mL^−1^) was mixed with 30 mL of distilled water in a 250-mL flask. The substrates of GQD were subjected to autoclaving at 121 °C for 20 min, then shook evenly and allowed to cool naturally. The sterilized substrates of GQD were inoculated with 2% (v/v) recovered SC and incubated at 28 °C in a shaking incubator (180×*g*). GQD samples were fermented for 48 h and then evaporated to dryness.

The concentrations of GQD and FGQD were approximately 2 g mL^−1^ (crude drug per g mL^−1^) for the animal experiments. In addition, the GQD and FGQD extracts for LC and LC–MS analysis were also prepared using the same protocol mentioned above in triplicate.

### Animal handing and biochemical parameters related to T2DM measurement

Male Sprague–Dawley rats (200–220 g) were purchased from Beijing Vital River Laboratories Co., Ltd. (SCXK (Jing) 2014-0013, Beijing, China). The rats were housed at a controlled room temperature of 23 ± 2 °C, 55 ± 10% humidity and a 12-h dark–light cycle for 10 days with free access to food and water. Then, 70 rats were randomly divided into two groups: the normal control group (NC, n = 10) and the diabetic rats group (n = 60). The NC group was fed a regular diet. The diabetic rats group was fed a high-sugar and HFD containing 5% sucrose, 10% lard, 5% yolk powder, 1% cholesterol, 0.1% sodium cholate and 78.9% regular diet. After 4 weeks of dietary intervention, the diabetic rats were fasted for 24 h and then received STZ (35 mg kg^−1^) dissolved in citrate buffer (0.1 M, pH 4.5) by intraperitoneal injection. The rats in the NC group received an equivalent volume of citrate buffer vehicle. One week after injection, fasting blood glucose (FBG) levels were determined using a drop of blood from the tail vein. Rats with FBG level above 11.1 mM were randomly subdivided into four groups (n = 13 for each group): the diabetic model group (DM) and three treatment groups. The treatment groups were fed 0.67 mg kg^−1^ of metformin hydrochloride (HM), 20 g kg^−1^ of GQD, or 20 g kg^−1^ of FGQD (crude drug per g kg^−1^ of body weight) every day for 8 weeks. Body weights were recorded every week, and FBG levels were measured every 2 weeks throughout the experiment.

At the end of the experimental period, the rats were sacrificed under anaesthesia, and blood was immediately collected. Total serum cholesterol (TC), triglycerides (TG), high-density lipoprotein cholesterol (HDL-C) and low-density lipoprotein cholesterol (LDL-C) levels were measured by an ELISA kit (Nanjing jiancheng Bioengineering Institute, Nanjing, China). The fast serum insulin (FINS) concentration was measured using commercial kits (Wa Lan Biotechnology, Shanghai, China). The insulin sensitivity index (ISI) was calculated according to FBG and FINS. The following formula for ISI was used: Ln (1/FBG * FINS) [24]. Homeostasis model assessment-insulin resistance (HOMA-IR) was calculated to measure the insulin sensitivity of the rats fed the experimental diets using the following formula: [FINS × FBG] 22.5^−1^ [25].

### Statistical analysis

Data are expressed as the mean ± S.D. All grouped data were statistically analysed with SPSS 13.0. Statistical significances between means were determined using one-way ANOVA followed by the LSD test of variance homogeneity and Dunnett’s T3 test of variance heterogeneity after the normal distribution test. Unless otherwise specified, a value of *p *< 0.05 was selected for discriminating significant differences throughout.

### Preparation of standard and sample solutions for HPLC–MS and HPLC analysis

For HPLC quantification, a mixed stock solution of ten reference substances was prepared at concentrations ranging from 1.0 to 2.5 mg mL^−1^ in 70% methanol. A standard working solution of the mixtures was obtained by diluting the stock solutions to the desired concentrations. All solutions were stored at 4 °C before use.

To obtain sufficient chemical ingredients in the GQD and FGQD extracts, dried extracts (0.1 g) were accurately weighted and separately extracted in 25 mL of 70% methanol (v/v) for 30 min by ultrasonication. After adjustment to the initial weight with methanol, 1 µL and 10 µL of the supernatant solution (obtained by centrifuging at 13,000×*g* for 10 min) were subjected to LC–MS and LC analysis, respectively. To validate the stability of the sample preparation and instrument, a pooled sample of all samples was prepared as quality control samples (QCs) for LC–MS. QCs were injected six times before the batch process and injected one time every 12 samples during the analysis process.

### Untargeted metabolomics analysis by HPLC Q Exactive MS

An HPLC Ultimate™ 3000 instrument coupled with a Q Exactive MS (Thermo Scientific, Bremen, Germany) was used for untargeted metabolomics in this study. Chromatographic separation was performed on an Agilent Poroshell 120 EC-C_18_ column (3 × 100 mm, 2.7 µm, Agilent, CA, USA). The mobile phase consisted of water containing 0.1% (*v*/*v*) formic acid (A) and acetonitrile (B). The following gradient was used: 0–10 min, 5% B to 17% B; 10–12 min, 17% B; 12–14 min, 17% B to 22% B; 14–19 min, 22% B; 19–29 min, 22% B to 32% B; 29–30 min, 32% B to 50% B; 30–34 min, 50% B to 90% B. The column was equilibrated for 5 min prior to each analysis. The flow rate was 0.3 mL min^−1^, and the column temperature was maintained at 30 °C. The mass spectrometer was operated in both positive and negative ESI full MS–dd-MS/MS acquisition mode with the use of the following parameter settings: spray voltage, 3.5 kV; sheath gas: 35 arbitrary units; auxiliary gas: 10 arbitrary units; capillary temperature: 320 °C; S lens RF level: 55; heater temperature: 300 °C. Full scan data were recorded for ions with *m/z* 100–1500 at a resolution of 70,000 (FWHM defined at *m/z* 200) in profile format. The automatic gain control (AGC) target values were set at 1 × e^6^ and 3 × e^6^ ions, respectively. The injection time was set to 250 ms in ESI^−^ mode and 100 ms in ESI^+^ mode. The MS/MS event was triggered when the given precursor ion was detected in an isolation window of *m/z* 2.0. The stepped normalized collision energies (NCE) of the analytes were 10, 30 and 50.

### Targeted quantification analysis by HPLC

Targeted metabolite quantification was performed on a Waters ACQUITY UPLC H-Class system (Milford, MA, USA). Samples were separated on an Agela-MP C_18_ column (2.1 mm × 250 mm, 5 μm, Agela, Tianjin, China) maintained at 30 °C. The binary mobile phase consisted of water containing 0.1% formic acid (A) and acetonitrile (B) at a flow rate of 1.0 mL min^−1^. The optimized gradient elution program was set as follows: 5–20% B (0–25 min), 20% B (25–30 min), 20–22% B (30–35 min), 22–40% B (35–55 min), 40–63% B (55–65 min), 63–80% B (65–70 min). The UV signals from two separate channels of 254 nm and 276 nm were recorded.

### Data processing and analysis

Data from the HPLC Q Exactive MS acquisition and processing were used for chemical profile analysis using Xcalibur™ 2.2 (Thermo Fisher). The untargeted metabolomics analysis was conducted by using Compound Discovery (version 1.2.1, Thermo SCIEX), and the detailed workflow is shown in Additional file 2: Figure S1. The multivariate data matrix was introduced into SIMCA-P (Version 13.0, Umetrics AB, Umea, Sweden) software for “unsupervised” principal component analysis (PCA) and “supervised” orthogonal projection to latent structure-discriminant analysis (OPLS-DA). All variables were UV-scaled for PCA and Pareto-scaled for OPLS-DA.

## Results

### Antidiabetic effect

**Fig. 1:**
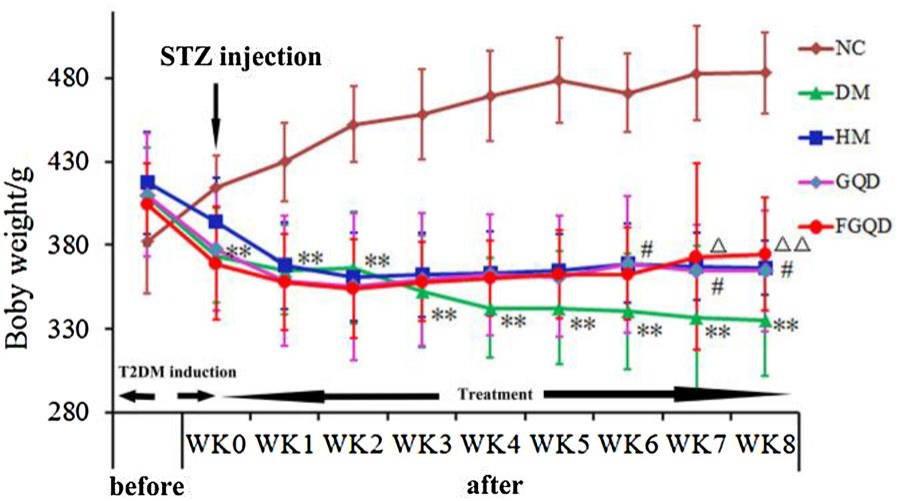
Effects of HM, GQD and FGQD on the body weight of T2DM rats. **p  < 0.01 DM vs NC; ^#^*p * < 0.05 HM vs DM; ^△^*p * < 0.05; ^△△^*p * < 0.01 FGQD vs DM

**Fig. 2:**
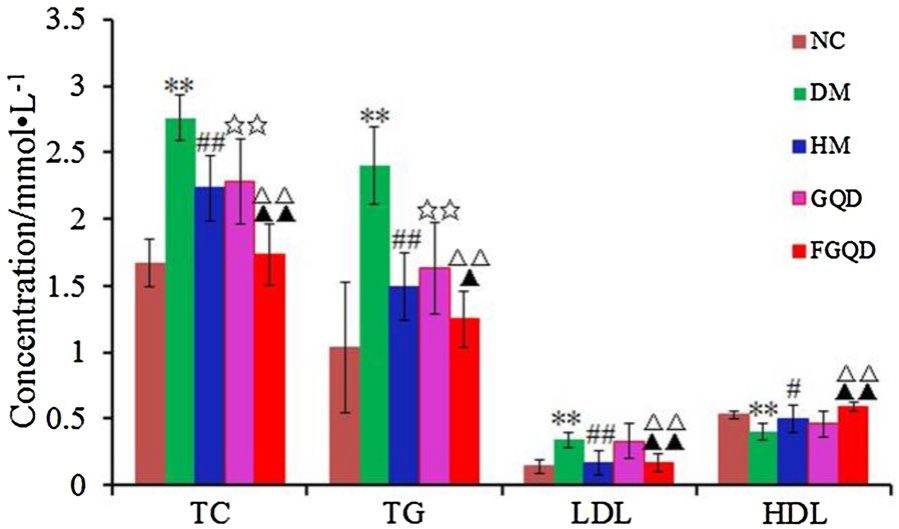
Effects of HM, GQD and FGQD on the serum lipid profile in T2DM rats. **p  < 0.01 DM vs NC; ^#^*p * < 0.05, ^##^*p * < 0.01 HM vs DM; ^☆☆^*p * < 0.01 GQD vs DM; ^△△^*p * < 0.01 FGQD vs DM; ^▲▲^*p * < 0.01 FGQD vs GQD

As shown in Fig. 1, the body weight of the diabetic rats decreased significantly compared with the NC group after STZ injection (*p *< 0.01). HM reversed the diabetes-induced body weight decrease from the 6th week (*p *< 0.05), whereas FGQD significantly reversed the body weight decrease from the 7th and 8th weeks (*p *< 0.01*, p *< 0.05). However, no significant (*p *> 0.05) effect was observed for the GQD group, suggesting that GQD had no significant effect on weight gain. As shown in Additional file 2: Figure S2, the FBG level was significantly increased in the diabetic rats compared to the NC group (*p *< 0.01) and was decreased in all drug-treated groups from the 4th week (*p *< 0.01*, p *< 0.05) after the injection of STZ. Although no significant difference was observed among the drug-treated groups (*p *> 0.05), the diabetic rats in FGQD showed a better trend of recovery. Rats in the model group had significantly higher levels of TC and TG (*p *< 0.01) than those in the NC group, and these levels were reduced in all drug-treatment groups (*p *< 0.01) (Fig. 2). Notably, the levels of TC and TG were significantly lower in the FGQD group than in the GQD group (*p *< 0.01) (Fig. 2). In addition, the treatments with HM and FGQD reversed the up-regulation of LDL and down-regulation of HDL in the diabetics rats group to the control level, whereas no significant (*p *> 0.05) effect was observed for GQD (Fig. 2). As shown in Table 1, the diabetic rats showed significant increases in FINS and HOMA-IR (*p *< 0.01) and a decrease in ISI (*p *< 0.01) compared with the NC group. After 8 weeks of drug administration, the levels of FINS, ISI and HOMA-IR were reversed compared with the DM group (*p *< 0.01). In addition, a notable difference in FINS level was observed in the FGQD group (*p *< 0.01) compared with the GQD group. In short, the body weight gain and the regulation of the levels of FINS, TC, TG, LDL and HDL in the FGQD group were significantly better than those in the GQD group (*p *< 0.01), but there were no significant differences in FBG, ISI and HOMA-IR levels between GQD and FGQD. These results suggested that FGQD had better therapeutic effect against diabetes than GQD.

**Table 1:** Effects of HM, GQD and FGQD on FINS, ISI and HOMA-IR of T2DM rats

Group	FINS (mIU/L)	ISI	HOMA-IR
NC	4.92 ± 0.74	− 3.38 ± 0.24	1.33 ± 0.30
DM	9.88 ± 0.58**	− 5.24 ± 0.22**	8.59 ± 1.75**
HM	7.17 ± 0.54^##^	− 4.47 ± 0.36^##^	3.98 ± 1.07^##^
GQD	6.78 ± 0.35^☆☆^	− 4.52 ± 0.23^☆☆^	4.18 ± 0.95^☆☆^
FGQD	5.86 ± 0.55^△△▲▲^	− 4.26 ± 0.18^△△^	3.20 ± 0.60^△^

*NC* normal control, *DM* diabetic model, *HM* metformin hydrochloride, *ISI* insulin sensitivity index, *FINS* fast serum insulin, *HOMA-IR* homeostasis model assessment-insulin resistance, *T2DM* type 2 diabetes mellitus

**p < 0.01 DM vs NC; ^#^*p *< 0.05, ^##^*p *< 0.01 HM vs DM; ^☆☆^*p *< 0.01 GQD vs DM; ^△△^*p *< 0.01 FGQD vs DM; ^▲▲^*p *< 0.01 FGQD vs GQD

### Characterization of the chemical constituents in the GQD extract

Since herbal medicines are generally taken as a decoction, we focused on boiled water extracts of GQD and their fermentation. The structural characterization of compounds in GQD is an essential step in identifying and matching those compounds with their secondary metabolites obtained through biotransformation. All known compounds were identified by comparison with chemical standards. For unknown compounds, structures were tentatively characterized based on retention time and MS spectra by referring to the previous literature. Finally, assignments of all compounds were further conducted by comparing the corresponding extracted ion chromatography (EIC) of GQD with those of the individual herbs. In total, 133 compounds were rapidly identified or tentatively characterized; these compounds were divided into six structural types. The detailed information, including retention times, accurate *m/z*, ppm errors, characteristic fragment ions, identified names and formulas, are summarized in Table 2, Additional file 2: Figure S3. Notably, two compounds were identified for the first time in GQD: 6-d-xylose-genistin and kuzubutenolide A.

**Table 2:** Retention time (*t*_*R*_), and MS data for identification of 133 compounds in GQD by HPLC Q Exactive MS

Source	*t*_R_ (time)	Compound	Formula	Experimental *m/z*	Error ppm	Mode	MS/MS (*m*/*z*)	Structure type
P1	6.85	3’-Hydroxypuerarin-4’-*O*-glucoside	C_27_H_30_O_15_	595.16559	− 0.263	+	475, ***433***, 415, 397, 379, 367, 337, ***313***, ***283***	*C*-glycoside-*O*-glu
P2	7.19	Puerarin-4’-*O*-glucoside	C_27_H_30_O_14_	579.17120	0.636	+	417, 399, 381, 351, 321, ***297***, ***267***, 255	*C*-glu-*O*-glu
P3	7.80	3’-Methyoxy puerarin-4’-*O*-glucoside	C_28_H_32_O_15_	609.18237	1.598	+	447, 429, 411, 393, 381, 365, 351, 327, ***297***	*C*-glycoside-*O*-glu
P4	8.03	Mirificin-4’-O-*β*-D-glucoside	C_32_H_38_O_18_	709.19830	0.859	−	457, 429	*C*-glycoside-*O*-glu
P5	8.33	Daidzein-4’,7-*O*-glucoside	C_27_H_30_O_14_	579.17084	0.014	+	417, ***255***	*O*-glu
P6	8.77	3’-Hydroxypuerarin*	C_21_H_20_O_10_	433.11276	− 0.377	+	415, 397, 379, 367, 337, ***313***, ***283***	*C*-glu
P7	8.89	3’-Methoxy-4’-*O*-glucoside-daidzin	C_28_H_32_O_15_	609.18262	2.008	+	447, 285	*O*-glu
P8	9.63	3’-Hydroxypuerarin xyloside	C_26_H_28_O_14_	565.15509	− 0.163	+	433, ***415***, 397, 379, ***367***, 337, ***313***, ***283***	*C*-glycoside-*O*-xyl
C1	10.07	Dihydro-11-Hydroxy-stepholidine-glucoside	C_25_H_31_NO_10_	506.20200	− 0.073	+	344, 326, 295, 277	alkaloid-*O*-glu
P9	10.30	6’’-O-α-D-glucopyranosylpuerarin	C_27_H_30_O_14_	579.17059	− 0.242	+	417, 399, 381, 351, 321, ***297***, 267	*C*-glu-*O*-glu
P10	10.43	3’-Hydroxydaidzin	C_21_H_20_O_10_	433.11389	0.967	+	271	*O*-glu
P11	10.62	Puerarin*	C_21_H_20_O_9_	417.11786	− 0.356	+	399, 381, 363, 351, 321, ***297***, ***267***, 255	*C*-glu
P12	10.73	Mirificin	C_26_H_28_O_13_	549.16016	− 0.107	+	417, 399, 363, 351, 321, ***297***, ***267***	*C*-glycoside-*O*-api
P13	11.16	3’-Methoxypuerarin	C_22_H_22_O_10_	447.12827	− 0.678	+	429, 411, 381, 351, 327, ***297***	*C*-glu
P14	11.22	6’’-*O*-Xylosylpuerarin	C_26_H_28_O_13_	549.15997	− 0.541	+	417, 399, 363, 351, 321, ***297***, ***267***	*C*-glycoside-*O*-xyl
C2	11.52	Magnoflorine*	C_20_H_23_NO_4_	342.16996	− 0.189	+	297, 265, 250, 237	alkaloid
P15	11.65	3’-Methoxypuerarin6’’-*O*-D-api	C_27_H_30_O_14_	579.17053	− 0.521	+	447, 429, 411, 393, 381, 365, 351, 327, ***297***	*C*-glu
C3	12.03	Norisocorydine	C_19_H_22_NO_4_	328.15411	− 0.225	+	313, 298, 282	alkaloid
S1	12.11	2’,3,5,6’,7-Pentahydroxyflavanone	C_15_H_12_O_7_	303.05096	1.031	−	285, 275, 217, 177	flavanones aglycone
P16	12.15	3’-Methoxydaidzin 6’’-*O*-D-api	C_27_H_34_O_11_	579.17108	0.428	+	255	*O*-glu
P17	12.19	5’-Hydroxypuerarin	C_21_H_20_O_10_	433.11328	0.824	+	415, 397, 367, ***313***, ***283***	*C*-glu
P18	12.57	Daidzin*	C_21_H_20_O_9_	417.11807	0.147	+	***255***	*O*-glu
C4	12.75	11-Hydroxy-stepholidine-glucoside	C_25_H_30_NO_10_	505.18610	− 0.322	+	342, 324, 275	alkaloid-*O*-glu
P19	13.02	Genistein-8-*C*-xyl-glucoside	C_26_H_28_O_14_	565.15619	1.008	+	433, ***415***, 397, ***367***, ***313***, ***283***	*C*-glycoside *O*-xyl
P20	13.40	BiochaninA-7-*O*-glucoside	C_22_H_22_O_10_	447.12933	1.692	+	285, 270, 253, 225	*O*-glu
C5	13.61	*O,O*’-Dimethoxyl magnoflorine	C_21_H_25_NO_5_	372.18045	− 0.099	+		alkaloid
P21	13.72	Genistein-8-*C*-api-glucoside	C_26_H_28_O_14_	565.15503	− 0.269	+	433, ***415***, ***367***, 337, ***313***, ***283***	*C*-glycoside-*O*-api
P22	14.48	PuerosideA	C_29_H_34_O_14_	607.20148	− 0.652	+	592, 461, 299, 281, 253	*O*-glu
P23	14.53	Daidzein 4’-*O*-glucoside	C_21_H_20_O_9_	417.11771	− 0.229	+	***255***, 199	*O*-glu
G1	14.79	Liquiritengin-glucopyranoside-(1→2)-β-D apiofuranoside	C_26_H_30_O_13_	549.16101	1.352	−	255, 153, 135, 119	*O*-glu-*O*-api
S2	14.96	Chrysin-6-*C*-pen-8-*C*-hex	C_26_H_28_O_13_	547.14851	2.180	−	487, 457, ***427***, 367, 337, 281	*C*-glu
S3	15.00	Viscidulin I	C_15_H_10_O_7_	301.03522	1.241	−	283, 273, 257, 229, 193, 151	flavone aglycone
C6	15.10	13-Hydroxyepiberberine	C_20_H_17_NO_5_	352.11787	− 0.149	+	336, 322, 294	alkaloid
S4	15.36	Chrysin-6-*C*-pen-8-*C*-hex	C_26_H_28_O_13_	547.14600	2.527	−	487, 457, ***427***, 367, 337, 281	*C*-glu
G2	15.38	Liquiritin apioside	C_26_H_30_O_13_	549.16046	0.351	−	***255***, 153, 135, 119, 91	*O*-glu
G3	15.44	Liquiritin*	C_21_H_22_O_9_	417.11917	2.784	−	***255***, 153, 135, 119	*O*-glu
S5	15.83	Chrysin 6-*C*-α-L-arabinoside-8-*C*-β-D-glucoside	C_26_H_28_O_13_	547.14581	2.18	−	487, 457, 427, 367, 337, 281	*C*-glu
P24	15.90	Neopuerarin	C_21_H_20_O_9_	417.11755	− 1.100	+	399, 381, 363, 351, 321, ***297***, ***267***	*C*-glu
P25	15.97	6-D-xylose-Genistin	C_26_H_28_O_14_	565.15768	2.408	+	433, ***271***	*O*-glu
S6	16.14	Scutellarein 7-*β*-D-glucuronoside	C_21_H_18_O_12_	461.07266	2.619	−	285, ***267***	*O*-gluA
S7	16.16	Chrysin 6-*C*-β-L-arabinoside-8-*C*-β-D-glucoside	C_26_H_28_O_13_	547.14587	2.290	−	487, 457, 427, 367, 337, 281	*C*-glu
C7	16.28	Stecepharine	C_21_H_25_NO_5_	372.18015	− 0.399	+	222, 207, 189	alkaloid
S8	16.31	Viscidulin III 2’*-O-*glucoside	C_23_H_24_O_13_	507.11469	2.707	−	345, 330, 315	*O*-glu
S9	16.43	Acteoside	C_29_H_36_O_15_	623.19861	2.509	−	461, 161, 179	*O*-glu
P26	16.44	Genistin*	C_21_H_20_O_10_	433.11334	0.417	+	***271***	*O*-glu
P27	16.46	Kuzubutenolide *A*	C_23_H_24_O_10_	461.14017	− 4.053	+	299, 281, 253, 239	*O*-glu
S10	16.52	Chrysin 6-*C*-β-D-glucoside- 8-C-α-L-arabinoside	C_26_H_28_O_13_	547.14569	1.961	−	457, 427, 367, 337, 321	*C*-glu
P28	16.74	Formononetin-8-*C*-glucoside-*O*-api	C_27_H_30_O_13_	563.17694	1.023	+	431, 413, 311, 281	*C*-glu-*O*-api
C8	16.88	Groenlandicine	C_19_H_15_NO_4_	322.10712	− 0.821	+	307, 279	alkaloid
C9	16.99	Demethyleneberberine	C_19_H_17_NO_4_	324.12283	-0.205	+	308, 266, 281	alkaloid
S11	17.02	chrysin6-hexosyl-8*-C-*pentosyl	C_26_H_28_O_13_	547.14612	1.503	−	457, 427, 367, 337, 321	*C*-glu
P29	17.05	Formononetin-8-*C*-glucoside-*O*-xyloside	C_27_H_30_O_13_	563.17554	− 0.670	+	431, 413, 311, 281	*C*-glu
P30	17.10	6′′-*O*-Malonyl daidzin	C_24_H_22_O_12_	503.11804	− 0.362	+	***255***	*O*-glu
S12	17.19	Chrysin 6-*C*-β-D-glucoside-8-*C*-β-L- arabinoside	C_26_H_28_O_13_	547.14575	2.070	−	457, 427, 367, 337, 321	*C*-glu
P31	17.22	4′-Methoxypuerarin	C_22_H_22_O_9_	431.13364	− 0.043	+	413, 395, 377, 335, 311, 281	*C*-glu
S13	17.34	5,2′,6′-Trihydroxy-7,8-dimmethoxy flavone -2′-glucoside	C_23_H_24_O_12_	491.11948	2.194	−	329, 314, 299	*O*-glu
S14	17.36	Isoacteoside	C_29_H_36_O_15_	623.19843	1.383	−	461, 161, 179	*O*-glu
C10	17.38	Oxyberberine	C_20_H_17_NO_5_	352.11789	− 0.059	+	337, 336, 322, 308, 294	alkaloid
C11	17.64	Oxidated palmatine	C_21_H_21_NO_5_	368.14893	− 0.314	+	352, 336	alkaloid
G4	18.06	Pyrroside B	C_26_H_30_O_14_	565.15649	2.315	−	***271***, 151	*O*-glu
G5	18.59	5-Hydroxylliquiritin	C_21_H_22_O_10_	433.11404	1.117	−	***271***, 151, 119	*O*-glu
S15	19.12	5,7,2′,6′-Tetrahydroxyflavone	C_15_H_10_O_6_	285.04050	3.984	−	241, 199, 133, 151	flavone aglycone
P32	19.21	6′′-O-Acetyl daidzin	C_23_H_22_O_10_	459.12839	− 0.183	+	***255***	*O*-glu
C12	19.49	Columbamine	C_20_H_19_NO_4_	338.13849	− 0.576	+	323, ***308***, 294	alkaloid
S16	19.58	5,7,2′-Trihydroxy-6-methoxyflavone 7-*O*-glucuronide	C_22_H_20_O_12_	475.08975	0.848	−	299, 284, 175, 113	*O*-gluA
C13	19.70	Epiberberine	C_20_H_18_NO_4_	336.12274	− 0.876	+	321, 320, 292	alkaloid
C14	19.98	Coptisine*	C_19_H_13_NO_4_	320.09174	0.017	+	292, 262	alkaloid
C15	20.13	Jatrorrhizine	C_20_H_19_NO_4_	338.13855	− 0.398	+	323, ***308***, 294	alkaloid
P33	20.81	Sophoraside A or isomer	C_24_H_26_O_10_	473.14496	0.737	−	311, 267, 252	*O*-glu
G6	20.89	Isoliquiritin apioside	C_26_H_30_O_13_	549.16150	0.191	−	***255***, 153, 135, 119, 91	*O*-glu
S17	21.68	Scutellarein*	C_15_H_10_O_6_	285.04071	4.720	−	267, 239, 166, 137, 117	flavone aglycone
P34	22.04	Ononin*	C_22_H_22_O_9_	431.13361	− 0.101	+	***269***	*O*-glu
G7	22.13	Licuraside	C_26_H_30_O_13_	549.16040	0.133	−	255, 153, 135, 119	*O*-glu
G8	22.23	Isoliquiritin*	C_21_H_22_O_9_	417.11948	1.471	−	***255***, 153, 135, 119	*O*-glu
S18	22.44	Baicalein 7-β-D-glucoside	C_21_H_20_O_10_	433.11276	− 0.163	+	***271***	*O*-glu
S19	22.50	Baicalin*	C_21_H_18_O_11_	445.09216	0.028	−	***269***, ***241***, 223, 175, 113	*O*-gluA
S20	22.60	Eriodictyol	C_15_H_12_O_6_	287.05630	1.285	−	218, 161, 125	flavanones aglycone
C16	22.65	Worenine+CH2+2H	C_21_H_21_NO_4_	352.15433	− 0.285	+	334, 320	alkaloid
P35	23.45	Daidzein*	C_15_H_10_O_4_	255.06509	− 0.374	+	227, 199, 181, 153	flavanones aglycone
G9	23.47	Neoisoliquiritin	C_21_H_22_O_9_	417.11954	1.531	−	255, 153, 119	*O*-glu
C17	23.56	Worenine	C20H15NO4	334.10721	− 0.174	+	319, 306, 291	alkaloid
G10	23.59	Licochalcone B	C_16_H_14_O_5_	285.07687	3.929	−	270, 253, 191, 150	chalcones aglycone
G11	24.13	Licorice glycosideB	C_35_H_36_O_15_	695.19727	0.223	−	549, 531, 399, 255	*O*-glu
G12	24.18	Liquiritigenin*	C_15_H_11_O_4_	255.06641	4.801	−	237, 153, 135, 119, 91	flavanones aglycone
P36	24.21	Isoononin	C_22_H_22_O_9_	431.13461	0.951	+	***269***	*O*-glu
C18	24.53	Palmatine*	C_21_H_21_NO_4_	352.15417	− 0.468	+	337, 336, 322, 308, 294	alkaloid
P37	24.69	BiochaninA	C_16_H_12_O_5_	283.06110	3.533	−	268, 240, 211	flavone aglycone
P38	24.82	Apigenin*	C_15_H_10_O_5_	269.04568	1.230	−	241, 225, 213, 197	flavone aglycone
S21	24.88	Naringenin 7-O-β-D-glucuronide	C_21_H_20_O_11_	447.09454	2.352	−	271, 243, 113	*O*-gluA
C19	24.96	Berberine*	C_20_H_17_NO_4_	336.12274	− 0.876	+	321, 320, 306, 292	alkaloid
C20	25.27	Demethylcoptichine	C_30_H_25_NO_8_	528.16552	− 0.073	+	334, 319, 304	alkaloid
S22	25.52	Norwogonin-8*-O*glucuronide	C_21_H_18_O_11_	445.07779	2.814	−	***269***, ***251***, ***241***	*O*-gluA
S23	26.15	Trihydroxymethoxyflavone*-O*glucoside	C_22_H_22_O_11_	461.10977	1.932	−	299, 284, 283, 211, 173	*O*-glu
S24	26.18	Hydroxyl oroxylin A-7-*O*-glucuronide	C_22_H_20_O_12_	475.08813	1.028	−	***299***, ***284***	*O*-gluA
S25	26.40	4′-Hydroxylwogonin	C_16_H_12_O_6_	299.05606	1.0455	−	***271***, 227, 211, 165, 133	*O*-gluA
S26	26.57	Norwogonin-7-*O*glucuronide	C_21_H_18_O_11_	445.07748	2.117	−	***269***, ***251***, ***241***	*O*-gluA
S27	26.98	Chrysin-7-*O*-glucuronide	C_21_H_18_O_10_	431.09763	0.828	+	***255***	*O*-gluA
S28	27.00	Oroxylin A-7-*O*-glucuronide	C_22_H_20_O_11_	459.09357	3.001	−	***283***, ***268***, 175, 113, 85	*O*-gluA
S29	27.38	Hydroxyl wogonoside	C_22_H_20_O_12_	475.08810	0.998	−	***299***, ***284***	*O*-gluA
P39	27.49	Isoformononetin	C_16_H_12_O_4_	267.06650	1.315	−	252, 223, 199	isoflavone aglycone
C21	27.84	13-Methylberberine	C_21_H_19_NO_4_	350.13864	− 0.045	+	335, 334, 320, 318, 306	alkaloid
S30	28.09	Baicalein 6*-O-*glucuronide	C_21_H_18_O_11_	445.07791	3.083	−	***269***, ***241***, 225, 197	*O*-gluA
C22	28.16	Demethylcoptichine	C_30_H_25_NO_8_	528.16595	1.244	+	334, 319, 304	alkaloid
S31	28.52	Wogonoside*	C_22_H_20_O_11_	459.09348	2.815	−	***283***, ***268***, 175, 113, 85	*O*-gluA
S32	29.11	5,7-Dihydroxy-6,8-dimethoxyflavone-7-O glucuronide	C_23_H_22_O_12_	489.10565	2.898	−	313, 298, 283	*O*-gluA
P40	30.47	Genistein*	C_15_H_10_O_5_	269.04572	4.721	−	241, 225, 183, 159	isoflavone aglycone
S33	30.53	5,7,4′-Trihydroxy-8-methoxyflavone	C_16_H_12_O_6_	299.05637	4.532	−	***284***, 231, 136, 94	flavone aglycone
G13	31.48	Licorice saponin A3	C_48_H_72_O_21_	983.44867	0.435	−	821, ***351***	*O*-gluA-gluA
S34	31.88	Norwogonin	C_15_H_10_O_5_	269.04578	1.330	−	***251***, ***241***, ***223***	flavone aglycone
G14	32.14	22β-Acetoxylglycyrrhizic acid	C_44_H_64_O_18_	879.40295	2.341	−	***351***	*O*-gluA-gluA
S35	32.20	5,7,2′-Trihydroxy-6-methoxyflavone	C_16_H_12_O_6_	299.05630	1.285	−	***284***, 255	flavone aglycone
G15	32.19	Licorice saponin G2	C_42_H_62_O_17_	837.39185	1.819	−	***351***, 193	*O*-gluA-gluA
S36	32.19	Trihydroxydimethoxyflavone	C_17_H_14_O_7_	329.06674	3.528	−	314, 299	flavone aglycone
S37	32.40	Baicalein*	C_15_H_10_O_5_	269.06000	− 0.100	−	***251***, ***241***, ***223***, 213, 197	flavone aglycone
S38	32.64	Trihydroxy-methoxyflavone	C_16_H_12_O_6_	299.05627	4.198	−	***284***, 165, 137	flavone aglycone
G16	32.73	Isoliquiritigenin*	C_15_H_11_O_4_	255.06633	4.566	−	237, 153, 119, 91	chalcones aglycone
G17	32.80	Glycyrrhizic acid*	C_42_H_62_O_16_	821.39655	1.385	−	***351***	*O*-gluA-gluA
P41	32.97	Formononetin*	C_16_H_12_O_4_	267.06647	4.810	−	252, 223	isoflavone aglycone
G18	33.07	Glycyrrhizin isomer	C_42_H_62_O_16_	821.39642	1.227	−	***351***	*O*-gluA-gluA
G19	33.39	Licorice saponin C2	C_42_H_62_O_15_	805.40094	0.433	−	***351***, 193	*O*-gluA-gluA
G20	33.50	Licorice saponin B2	C_42_H_64_O_15_	807.41724	1.093	−	***351***, 193	*O*-gluA-gluA
S39	33.54	Skullcapflavone	C_18_H_16_O_7_	343.08255	1.321	−	328, 313, 298, 285	flavone aglycone
G21	33.72	Liconeolignan	C_21_H_22_O_5_	353.13947	1.120	−	338, 321, 295, 283, 269	others
S40	33.77	Wogonin*	C_16_H_12_O_5_	283.06140	4.593	−	***268***, ***239***, 163	flavone aglycone
S41	33.78	Chrysin	C_15_H_10_O_4_	255.06497	− 0.215	+	238, 214	flavone aglycone
S42	33.90	Dihydroxy-dimethoxyflavone	C_17_H_14_O_6_	315.08600	− 0.315	+	300, 285	flavone aglycone
C23	33.95	Berberastine	C_20_H_18_NO_5_	352.11774	− 0.209	+	336, 322, 308	alkaloid
S43	34.03	Oroxylin A	C_16_H_12_O_5_	283.06137	4.487	−	***268***, ***239***, 163	flavone aglycone
S44	34.21	Tenaxin I	C_18_H_16_O_7_	343.08252	1.321	−	268, 239, 163	flavone aglycone
G22	34.40	Licoisoflavone A	C_20_H_18_O_6_	353.10330	1.335	−	284, 267, 243, 216, 201, 83	isoflavone aglycone
G23	34.45	Licochalcone A	C_21_H_22_O_4_	337.14484	1.404	−	305, 281, 243, 229, 201	chalcones aglycone
G24	35.03	Glabrone	C_20_H_16_O_5_	335.09271	1.310	−	305, 291, 275, 213, 199, 107	isoflavone aglycone
G25	35.03	Licoisoflavone B	C_20_H_16_O_6_	351.08755	1.235	−	283, 265, 241, 199, 83	isoflavone aglycone

Major signals in MS spectra were indicated in bolditalic

*t*_*R*_ retention time, *P* Pueraria Lobatae Radix, *S* Scutellariae Radix, *C* Coptidis Rhizoma, *G* Glycyrrhizae Radix et Rhizoma Praeparata cum Melle, *+* detected in positive ion mode, *−* detected in negative ion mode, *confirmed with reference compounds

#### Isoflavone glycosides

In total, 17 isoflavone *C*-glycosides and 15 isoflavone *O*-glycosides were identified as the dominant compounds from Gengen in GQD (Additional file 2: Figure S4A). P6, P11, P18, P26 and P34 were unambiguously identified by comparison with reference compounds. According to the MS/MS analysis of these authentic compounds, isoflavone *O*-glycosides (P18, P26 and P34) showed dominant aglycone ions at *m/z* 255, 271 and 269, respectively, due to the loss of a glucose group (162 Da). By contrast, isoflavone *C*-glycosides (P6 and P11) were hardly cleaved under the same conditions and shared the common principal fission pattern of successive or simultaneous losses of CO, CHO and CH_2_O groups caused by cleavage of the *C*-ring. Consequently, the major fragmentation behaviours were summarized and then applied as rules to elucidate the structures of the other 27 unknown compounds with the same basic skeleton [18, 26, 27]. Among them, P25 showed a precursor ion with *m/z* 565.15509 and further fragmented into the characteristic ion at *m/z* 271, corresponding to [M+H–xyl/api–glu]^+^. More importantly, P25 was tentatively deduced as 6-d-xylose-genistin in GQD for the first time.

#### Flavone glycosides

The occurrence of flavone *O*-glucuronides is less common in plants. Previously published studies have thoroughly summarized the fragmentation pathways of flavonoids *O*-glucuronides in Huangqin [28]. As characteristic components, a total of 12 flavone *O*-glucuronides (S6, S16, S19, S22, S24, S26, S27, S28, S29, S30, S31 and S32) all from Huangqin were identified and tentatively characterized in GQD (Additional file 2: Figure S4B) [28–33]. Moreover, S2, S4, S5, S7, S10, S11 and S12 were tentatively characterized as flavone *C*-glycosides. In addition, S8, S13, S18 and S23 were excluded from flavone *O*-glucuronides by analysing the MS/MS spectra and then were finally identified as flavone *O*-glycosides [33].

In addition, six flavanones glycosides and five chalcones glycosides were putatively characterized in GQD (Additional file 2: Figure S4C). Among them, G3 and G8 were identified as liquiritin and isoliquiritin, respectively, by comparison with reference standards, and the others from Gancao were characterized by analysing their MS/MS spectra [32, 34]. In addition, S21 was characterized as a flavanone glycoside from Huangqin.

#### Free flavones

In total, 30 free flavones were tentatively assigned and could be further divided into isoflavones (8), flavones (16), flavanones (3) and chalcones (3) in GQD (Additional file 2: Figure S4D). P35, P40 and P41 were confirmed by comparison with reference standards. P37 and P39 from Gegen and G22, G24 and G25 from Gancao were tentatively characterized as isoflavone aglycones by analysing the MS/MS spectra [2, 32]. In addition, the flavones comprised 16 compounds from Huangqin. Baicalein (S37) produced characteristic ions with *m/z* 251, 241 and 223 by loss of H_2_O and CO. Wogonin (S40), a methoxylated flavonoid, presented a deprotonated ion [M−H]^−^ at *m/z* 283.06140 and characteristic fragment ions with *m/z* 268 and 239. In addition, a low signal intensity ion with *m*/*z* 163 (^0,2^A^−^) through Retro-Diels–Alder (RDA) cleavage was observed. Thus, the other 14 flavones in the complex mixtures were characterized based on the literature [28, 33]. In negative ion mode, liquiritigenin (G12) and isoliquiritigenin (G16), a pair of isomers, showed fragmentation patterns associated with RDA cleavage at *m/z* 135 or 119. Thus, S1, S20, G10 and G23 were tentatively characterized according to the above mentioned MS behaviours [28].

#### Alkaloids

A total of 23 alkaloids from Huanglian were characterized based on positive ion mode mass spectra (Additional file 2: Figure S4E). Three benzylisoquinoline alkaloids, i.e., coptisine, palmatine and berberine, were identified by comparison with their authentic standards and the production of one or multiple common small fragments such as H_2_O, CH_3_ and C_2_H_6_N, respectively. Based on these rules, C6, C8, C9, C10, C11, C12, C13, C15, C16, C21 and C23 were observed and further tentatively characterized by analysing characteristic ions [35, 36]. Magnoflorine, an aporphinoid alkaloid, exhibited a precursor ion at *m/z* 342.16996 and characteristic ions at *m/z* 297, 265, 250 and 237. Similarly, C4 and C5 were tentatively identified as aporphinoid alkaloids. The others (C1, C3, C7, C17, C20 and C22) were characterized by comparison to the literature [27].

#### Triterpene saponins

Triterpene saponins were the other characteristic constituents from Gancao. In total, six triterpene saponins were putatively identified (Additional file 2: Figure S4C). Glycyrrhizic acid (G17 or G18) presented an [M−H]^−^ ion with *m/z* 821.39655 and characteristic fragment ions at *m/z* 351 and 193 [32]. G13, G14, G15, G19 and G20 showed characteristic ions similar to those of glycyrrhizic acid and were tentatively characterized according to the literature [18].

#### Others

In addition to the major compounds described above, atypical structures were also found in GQD (Additional file 2: Figure S4C). P22 and P33, which belong to aromatic glycosides, were identified as pueroside A and sophoroside A or their isomers [26]. P27 showed an [M+H]^+^ ion at *m/z* 461.14017 with MS^2^ characteristic peaks at *m/z* 299, 281, 253 and 239 and was tentatively identified as kuzubutenolide A in GQD for the first time [37]. In addition, S9 and S14 were tentatively identified as isomers of acteoside and isoacteoside [12, 38], and P38 and G21 were also tentatively characterized by comparison with the literature [33].

### Multivariate statistical analysis

**Fig. 3:**
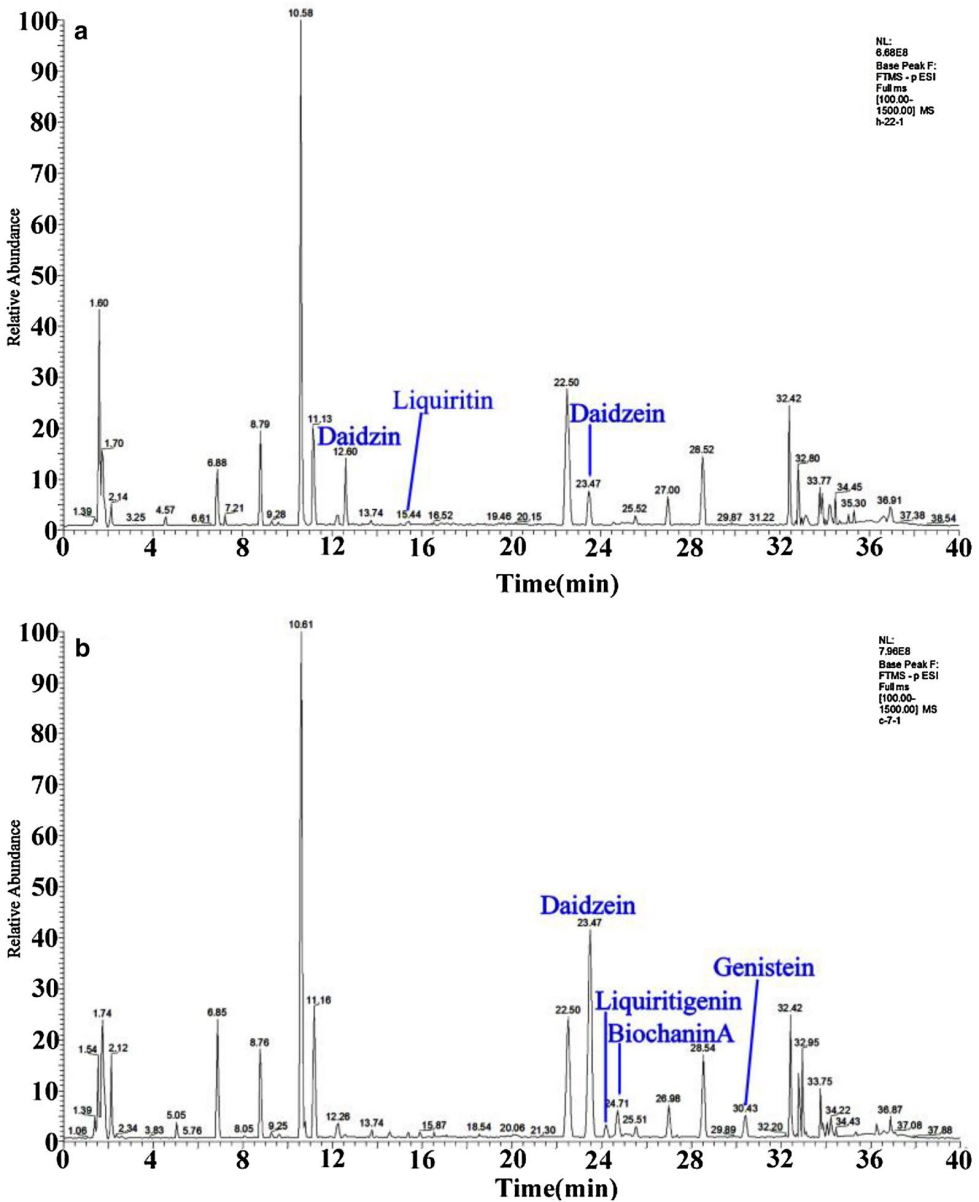
Typical basic peak ion chromatograms obtained by HPLC Q Exactive MS. **a** GQD; **b** FGQD. All chromatograms were obtained in negative ion mode

**Fig. 4:**
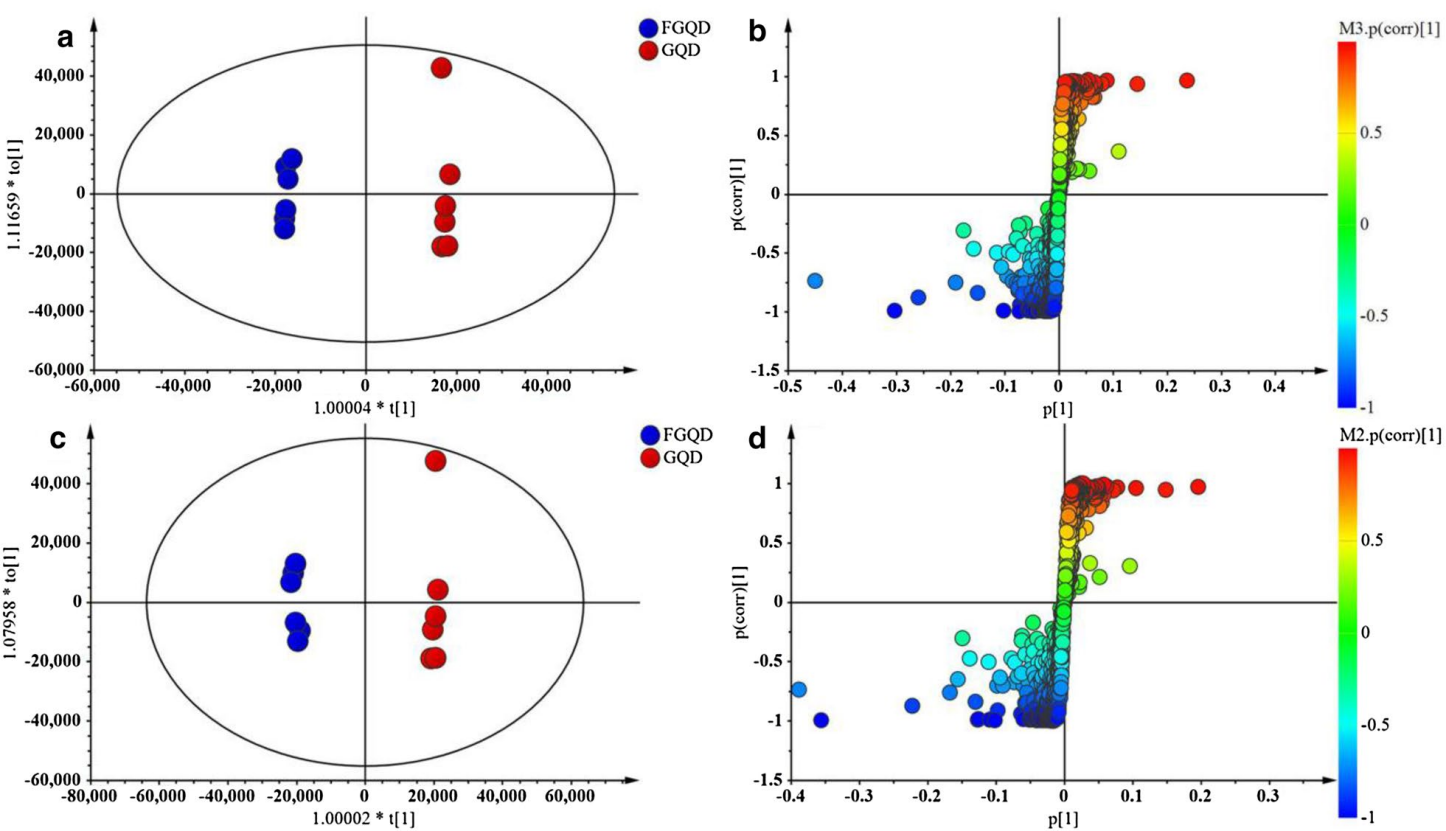
OPLS-DA score plots (**a**, **c**) and S-plots (**b**, **d**) between GQD and FGQD. **a** and **c** present data in positive ion mode; **b** and **d** present data in negative ion mode

To identify chemical markers distinguishing GQD and FGQD samples, the negative and positive ion mode data detected by HPLC Q Exactive MS were simultaneously used for global analysis. Visual inspection of the chromatograms for GQD and FGQD indicated that the fermentation process induced obviously different peak intensities; that is, FGQD contained more daidzein, liquiritigenin, genistein, and biochanin A and less daidzin and liquiritin than GQD (Fig. 3). Multivariate statistical analysis was subsequently applied to further reveal the minor differences between GQD and FGQD. In the PCA score plot (Additional file 2: Figure S5A, B) generated by PC1 (46.2%) and PC2 (17.9%) for positive ion mode and PC1 (51.1%) and PC2 (17.9%) in negative ion mode, clear separation can be observed between GQD and FGQD. Then, OPLS-DA was further performed to process the secondary metabolome data between the GQD and FGQD groups by S-plot and VIP-value analysis. The model fit parameters were 0.999 for R^2^Y (cum) and 0.971 for Q^2^ (cum) for positive ion mode and 0.999 for R^2^Y (cum) and 0.987 for Q^2^ (cum) for negative ion mode, respectively, suggesting that the OPLS-DA model exhibited good fitness and predictability. In the S-plots, each point represented an ion t_*R*_-*m/z* pair, whereas the distances of the pair points from the mean centre indicate the contribution of the variables in discriminating the GQD and FGQD groups (Fig. 4a, b). The VIP-value threshold cut-off of the variables was set to one, and thus 83 and 117 variables were finally screened in LC/MS (ESI^+^) and LC/MS (ESI^−^), respectively. Among them, 25 variables were identified in both ion modes. Three variables and two variables were identified in negative ion mode and positive ion mode, respectively. Thus, 30 compounds that had different intensities between GQD and FGQD were detected.

**Fig. 5:**
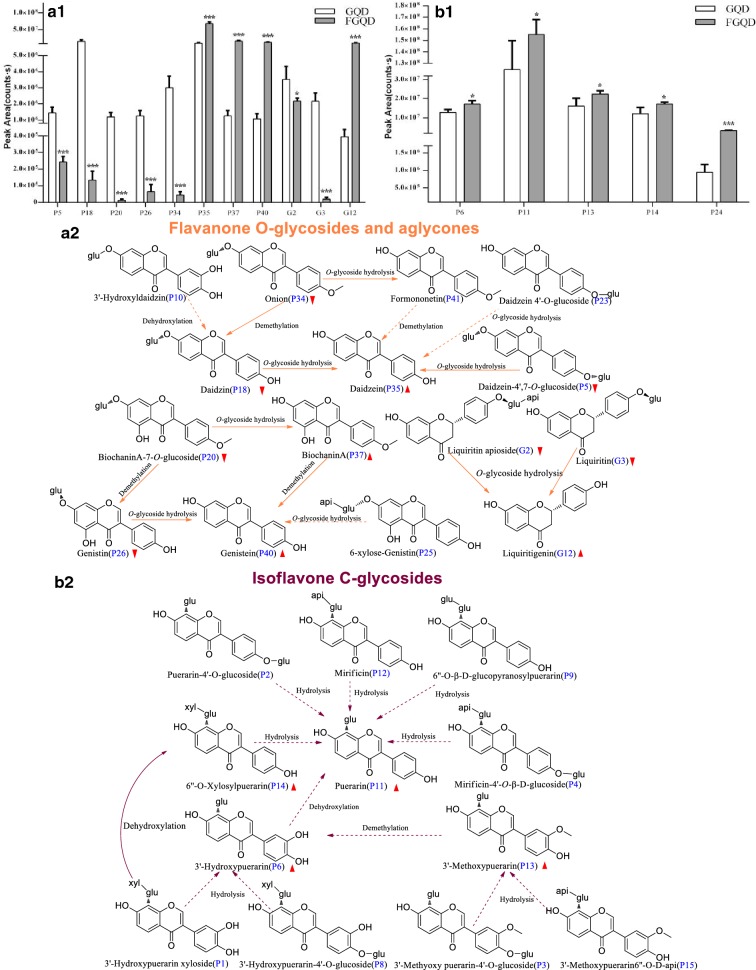
Proposed fermentation-induced chemical transformation mechanisms. **a1** Flavone *O*-glycosides and aglycones; **b1** isoflavone *C*-glycosides; **a2** proposed biotransformed pathways of flavone *O*-glycosides and aglycones; **b2** proposed biotransformed pathways of isoflavone *C*-glycosides. Solid arrows: prone to happen; dotted arrows: speculated/less likely to happen.  Indicates an elevation of the compound content;  Indicates a decrease in the compound content (***p < 0.001, *p < 0.05 GQD vs FGQD)

**Fig. 6:**
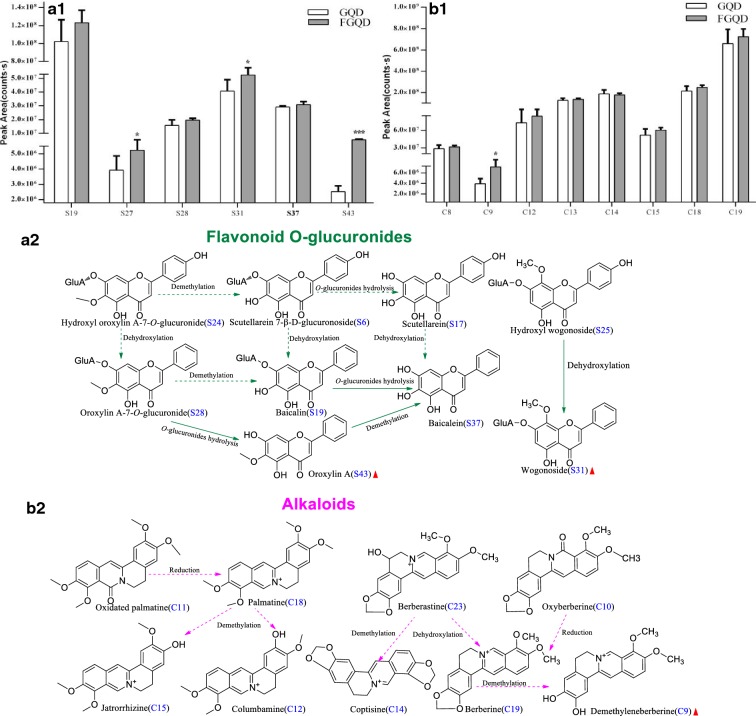
Proposed fermentation-induced chemical transformation mechanisms. **a1** Flavone *O*-glucuronides; **b1** alkaloids; **a2** proposed biotransformed pathways of flavone *O*-glucuronides; **b2** proposed biotransformed pathways of alkaloids. Solid arrows: prone to happen; dotted arrows: speculated/less likely to happen.  Indicates an elevation of the compound content;  Indicates a decrease in the compound content (***p < 0.001, *p < 0.05 GQD vs FGQD)

To maximize the understanding of the effect of fermentation on GQD, the mean peak areas and the t-test results for the significant differences in the 30 compounds from GQD and FGQD are shown in Figs. 5, 6. As shown in Fig. 5a1, the mean peak areas of free flavones (P35, P37, P40 and G12) were larger in FGQD than in GQD (*p *< 0.001), whereas the mean peak areas of their corresponding *O*-glycosides (P5, P18, P20, P26, G2 and G3) were smaller in FGQD than in GQD (*p *< 0.001, *p *< 0.05), indicating that *O*-glycoside hydrolysis occurred during fermentation processing (Fig. 5a2). P23 could also be transformed to P35 by *O*-glycoside hydrolysis. In addition, P10 and P34 contained abundant hydroxyl and methyl and were deduced to possibly produce P18 by dehydroxylation or demethylation. Actually, a marked decline in the level of P34 was also observed (*p *< 0.01) (Fig. 5a1), however, its corresponding aglycone P41 was not obviously altered in FGQD, which might be due to a dynamic equilibrium between their formation (from *O*-glycoside hydrolysis) and further transformation (e.g., demethylation). By contrast, *C*-glucosides appeared to be more difficult to transform by SC, since five *C*-glucosides (P6, P11, P13, P14 and P24) were detected in FGQD (Fig. 5b1). Their significant increasing trend was probably caused by the hydrolysis of low contents of puerarin *C*-glucoside-*O*-glucoside derivatives, such as P1, P2, P3, P4, P8, P12 and P15 (Fig. 5b2). *O*-*C* glycoside bonds have been reported to be the main effective target of β-glucosidase [13], in agreement with our results that puerarin (P11) and its derivatives were difficult to hydrolyse by *β*-glucosidase.

As shown in Fig. 6a1, the remarkable increase in the level of flavone aglycone (S43) was potentially due to hydrolysis of the corresponding flavone *O*-glucuronide (S28), which contains a 6-OCH_3_ group (*p *< 0.001). S31, which contains an 8-OCH_3_ group, was more difficult to transform by hydrolysis by SC but was easier to produce from S25 by dehydroxylation (Fig. 6a2). Although a different strain of yeast was used, the current findings are still in agreement with those in a previous study [39]. Notably, the increasing trend of S37 is likely partially responsible for the hydrolysis reactions of the corresponding compound (S19) (Fig. 6a2). A previous study demonstrated that *Escherichia* (*E.*) *coli β*-glucuronidases could hydrolyse glucuronic acid at the 7-position if the structure contains a 6-OH group [39]. Other metabolic reactions for flavone-*O*-glucuronides, including demethylation and dehydroxylation, were also deduced.

Due to the lack of a free hydroxyl group, alkaloids are demethylated to form free hydroxyl groups by SC [36]. In this study, a significant increase in demethyleneberberine (C9) was observed in FGQD compared to GQD (*p *< 0.05), which probably contributed to the demethylation of C19 during fermentation processing (Fig. 6b1, b2). There were no significant differences in the other benzylisoquinoline alkaloids between GQD and FGQD (*p *> 0.05), thus indicating that the contents of these molecules remained stable during the fermentation process.

### Targeted quantification analysis

As mentioned above, the untargeted metabolomic studies indicated that isoflavone *O*-glycosides, flavone *O*-glycosides, flavone *O*-glucuronides and alkaloids were potential chemical markers for distinguishing GQD and FGQD. Thus, three *O*-glycosides (daidzin, baicalin and liquiritin), one *C*-glycoside (puerarin), three flavones (daidzein, liquiritigenin, and baicalein), and three alkaloids (coptisine, berberine and palmatine) were quantitatively determined as examples to illustrate the effects of processing (Additional file 2: Figure S3, Table S1). Their content changes in GQD and FGQD are summarized in Table 3. As expected, fermentation processing significantly depleted liquiritin (*O*-glycoside) from 0.80 ± 0.06 mg g^−1^ to 0.48 ± 0.02 mg g^−1^ (*p *< 0.05), whereas daidzin was not even detectable in FGQD (*p *< 0.001) after fermentation with SC. Interestingly, the concentrations of daidzein and liquiritigenin (free flavones) in FGQD were greatly enhanced (*p *< 0.001, *p *< 0.05, respectively). In addition, an obvious increase in the level of puerarin (isoflavone *C*-glycoside) was observed until the end of fermentation. Regarding alkaloids, the contents of coptisine, palmatine and berberine remained relatively stable (*p *> 0.05). Moreover, there was a slight increasing trend for baicalin (flavone *O*-glucuronide), whereas no significant difference was found between GQD and FGQD. Interestingly, the quantitative results revealed an increasing trend for baicalein (*p *> 0.05) did not correspond to the results of the untargeted studies, which showed a significant increase in the content of baicalein in FGQD compared with GQD (*p *< 0.05).

**Table 3:** Contents of 10 chemical markers in GQD and FGQD by SC (mg g^−1^, n = 3)

Compound	0 h	48 h
Puerarin	20.30 ± 0.05	23.57 ± 0.02*
Daidzin	3.67 ± 0.08	n.d.***
Daidzein	0.50 ± 0.02	3.80 ± 0.01***
Liquiritin	0.80 ± 0.06	0.48 ± 0.02*
Liquiritigenin	0.17 ± 0.05	0.50 ± 0.01*
Coptisine	2.23 ± 0.12	2.68 ± 0.003
Palmatine	2.01 ± 0.03	2.36 ± 0.15
Berberine	7.70 ± 0.03	8.10 ± 0.02
Baicalin	10.80 ± 0.02	11.85 ± 0.01
Baicalein	1.26 ± 0.04	1.27 ± 0.03

The content is expressed in units of mg/g. n.d. indicates under the LOQ. *p < 0.05, ***p < 0.001 FGQD vs GQD

## Discussion

GQD is a well-known TCM formula that has been reported to display anti-diabetic properties in the clinic [20]. In the present study, we investigated the efficiency of FGQD and confirmed that fermentation actually enhanced the anti-diabetic activities of GQD in vivo in diabetic rats induced by HFD and STZ. The present results suggested that GQD had no significant effect on weight gain, in agreement with a previous study [19], whereas FGQD showed a significant reversed trend. In addition, our study indicated that the level of FBG was conspicuously decreased, accompanied by decreases in serum TG, TC, LDL-C and FINS and increased HDL-C after GQD treatment, consistent with previous work [21]. FGQD exerted greater regulatory effects on the levels of TC, TG, LDL-C, HDL-C and FINS compared to GQD. Thus, both GQD and FGQD displayed effects against HFD and STZ-induced diabetes, and FGQD showed a better recovery trend associated with profound changes in the serum lipoprotein profile and body weight gain. These findings further suggest that fermentation can play a key role in the search for therapeutically useful drugs. Given the pharmacologically decisive roles of the involved ingredients, chemical transformations might significantly contribute to the therapeutic differences between GQD and FGQD. Thus, the chemical profiles of GQD and FGQD were further systematically compared using the proposed integrated strategy based on untargeted and targeted metabolomic analysis.

In this study, 133 secondary metabolites analysed using UPLC-Q Exactive MS were identified and characterized by comparison with standard references and the literature. Then, untargeted metabolomics was performed to find statistically significant differences between GQD and FGQD groups via OPLS-DA S-plot and VIP-value analysis. The OPLS method is a modification of the PLS method with a multivariate pre-processing filter called orthogonal signal correction (OSC). The OSC filter removes uncorrelated signals to provide information on the within-class variation [40]. Overall, 30 potential chemical markers contributed to the separation of GQD and FGQD, and the mechanisms of the processing-induced chemical transformation of the secondary metabolites were further elucidated. Although there were no new secondary metabolites in FGQD compared with GQD, the amounts of these secondary metabolites were redistributed in FGQD. Deglycosylation reaction by stepwise cleavage of the sugar moieties was considered the main metabolic pathway. Other chemical reactions, i.e., dehydration, demethylation and reduction, were also potentially implicated in the processing. These chemical transformations should mainly contribute to the fluctuation in the contents of isoflavone *O*-glycosides and flavone *O*-glucuronides due to processing. These results for the in vitro biotransformation of GQD by SC demonstrated that the fermentation of TCM formulas is a complex process.

Due to the lack of reference standards for quantitation and poor baseline separation, only ten representative compounds with high contents were subjected to targeted analysis to illustrate the effects of processing. For puerarin, daidzin, daidzein, liquiritin and liquiritigenin, the results of the targeted quantification were consistent with those obtained in the untargeted studies, thus demonstrating that the hydrolysis of *O*-glycosides occurred due to the effect of *β*-glucosidase of SC [2, 41, 42] and further supporting speculation that *C*-glucoside is more difficult to transform via biotransformation with SC. In addition, the variation trends of coptisine, berberine, palmatine and baicalin in the targeted quantification corresponded with the results of the untargeted metabolomics, suggesting that multiple reactions might simultaneously occur, resulting in a dynamic equilibrium (Figs. 5, 6). Interestingly, the increasing trend of baicalein in the targeted analysis was highly different from the significant increase in baicalein observed in the untargeted analysis. Thus, we conclude that baicalein is altered slightly due to the dynamic equilibrium between flavone *O*-glucuronides and their derivatives. According to these results, our integrated strategy was useful for screening, matching and identifying the metabolites of FGQD.

Increasing evidence has indicated that the ten targeted compounds detected in raw and fermented GQD have various regulatory actions against T2DM. The anti-diabetic effects of Gegen isoflavones have been demonstrated in several studies [43–46]. A previous study showed that both puerarin and daidzein from Gegen could reduce FBG and improve ISI and hyperlipidaemia in diabetic mice or rats [43–45], whereas daidzin showed an opposite effect by stimulating glucose uptake [46]. In addition, it was reported that daidzein can improve plasma TC, TG and HDL-C concentrations in *db/db* mice [43]. Gaur reported that liquiritigenin from Gancao could be used as a possible lead for the control of FBG levels [47]. Several studies have shown that daidzein and liquiritigenin, which are small, hydrophobic molecules, are absorbed faster and in higher amounts than their glucosides, daidzin and liquiritin, in humans [44]. Thus, the increasing trends of flavone aglycones (daidzein and liquiritigenin) and isoflavone *C*-glycosides (puerarin), as well as other homologous compounds, might be helpful for explaining the greater anti-diabetic effects of FGQD, which occur partially via regulation of the levels of ISI, TC, TG, and HDL. Moreover, baicalin and baicalein from Huangqin have been demonstrated to exhibit excellent anti-diabetic activities [48–50]. Berberine, palmatine and coptisine have also been reported to exert antidiabetic effects involved in improving insulin resistance and secretion and promoting glucose consumption in 3T3-L1 murine pre-adipocytes cells [51–53]. Thus, the stable contents of baicalin, baicalein, coptisine, berberine and palmatine, which showed obvious antidiabetic effects, as well as other compounds in FGQD, may contribute to the observed anti-diabetic effects. Taken together, these findings will help enhance our understanding of the greater anti-diabetic effects of FGQD.

## Conclusions

In the present study, the antidiabetic effects and chemical profiles between GQD and FGQD were systematically compared. The anti-diabetic effects of FGQD were more potent than those of GQD, suggesting that the anti-diabetic activities of TCM formulas might be improved by applying fermentation technology. Moreover, the integration of chromatographic technique-based untargeted metabolomics and targeted analysis can be considered a useful approach for systematically exploring the chemical profiles of raw and fermented formulas. The increasing activities might be ascribed to the main constituents of transformation between GQD and FGQD. To ensure the therapeutic effects and safety of FGQD, the role of fermentation in processing should be further studied.

## Additional files

**Additional file 1.** Minimum standards of reporting checklist.

**Additional file 2: Table S1.** Calibration curves, LODs, LOQs, repeatability, accuracy and stability of the quantitative assays for 10 analytes in GQD. **Figure S1.** Workflow of the untargeted metabolomic analysis. **Figure S2** Effects of HM, GQD and FGQD on the FBG levels in T2DM rats. ^**^p<0.01 DM vs NC; ^#^*p*<0.05, ^##^*p*<0.01 HM vs DM; ^☆^*p*<0.05, ^☆☆^*p* <0.01 DM vs GQD; ^△^*p*<0.05, ^△△^*p* <0.05 FGQD vs DM. **Figure S3.** Chemical structures of the compounds identified in GQD. P: Pueraria Lobatae Radix; S: Scutellariae Radix; C: Coptidis Rhizoma; G: Glycyrrhizae Radix et Rhizoma Praeparata cum Melle. **Figure S4.** Extracted ion chromatograms of 133 constituents from GQD. P: Pueraria Lobatae Radix; S: Scutellariae Radix; C: Coptidis Rhizoma; G: Glycyrrhizae Radix et Rhizoma Praeparata cum Melle. **Figure S5.** PCA score plots of GQD and FGQD. A: negative ion; B: positive ion. **Figure S6.** Representative HPLC chromatograms of ten marker compounds at 254 nm and 276 nm. P11: puerarin, P18: daidzin, P35: daidzein, C14: coptisine, C18: palmatine, C19: berberine, G3: liquiritin, G12: liquiritigenin, S19: baicalin, S37: baicalein.

## Abbreviations

GQD: Ge-Gen-Qin-Lian decoction
FGQD: fermented Ge-Gen-Qin-Lian decoction
TCM: traditional Chinese medicine
SC: *Saccharomyces cerevisiae*
HPLC: high-performance liquid chromatography
MS: mass spectrometry
PD: potato dextrose
T2DM: type 2 diabetes mellitus
STZ: streptozotocin
NC: control group
HFD: high-fat diet
FBG: fasting blood glucose
HM: metformin hydrochloride
TC: total serum cholesterol
TG: triglycerides
HDL-C: high-density lipoprotein cholesterol
LDL-C: low-density lipoprotein cholesterol
FINS: fast serum insulin
HOMA-IR: homeostasis model assessment-insulin resistance
RDA: Retro-Diels–Alder
QCs: quality control samples
PCA: principal component analysis
OPLS-DA: orthogonal projection to latent structure-discriminant analysis
AGC: automatic gain control
NCE: normalized collision energies
EIC: extracted ion chromatography
