# Supplementary material for: Comparing the antidiabetic effects and chemical profiles of raw and fermented Chinese Ge-Gen-Qin-Lian decoction by integrating untargeted metabolomics and targeted analysis
Source: Chin Med. 2018 Oct 26;13:54. doi: 10.1186/s13020-018-0208-7 (PMC6204051; doi:10.1186/s13020-018-0208-7)

**Additional Information**

**Comparing the antidiabetic effects and chemical profiles of raw and fermented Chinese Ge-Gen-Qin-Lian decoction by integrating untargeted metabolomics and targeted analysis** Yan Yan^1a^, Chenhui Du^1b^, Zhenyu Li^a^, Min Zhang^a,c^, Jin Li ^b^, Jinping Jia^a^, Aiping Li^a^, Xuemei Qin^a*^, Qiang Song ^b*^

a *Modern Research Center for Traditional Chinese Medicine of Shanxi University, No. 92, Wucheng Road, Taiyuan 030006, Shanxi, China*

b *School of Traditional Chinese Materia Medica, Shanxi University of Chinese Medicine, No.121, Daxue Street, Taiyuan 030619, Shanxi, China*

c *College of Chemistry and Chemical Engineering of Shanxi University, No. 92, Wucheng Road, Taiyuan 030006, Shanxi, China*

*Corresponding author: Tel.: +86-351-7018379, +86-351-3179978; fax: +86-351-7011202, +86-351-3179978.

E-mail address: [qinxm@sxu.edu.cn](mailto:qinxm@sxu.edu.cn); sxhpe[@163.com](mailto:boyangyu59@163.com)

1 These two authors contributed equally to this work.

**Additional information available**

**Table S1** Calibration curves, LODs, LOQs, repeatability, accuracy and stability of the quantitative assays for 10 analytes in GQD.

**Figure S1** Workflow of the untargeted metabolomic analysis.

**Figure** S**2** Effects of HM, GQD and FGQD on the FBG levels in T2DM rats.

^**^*p*<0.01 DM vs NC; ^#^*p*<0.05, ^##^*p*<0.01 HM vs DM; ^☆^*p*<0.05, ^☆☆^*p* <0.01 DM vs GQD; ^△^*p*<0.05, ^△△^*p* <0.05 FGQD vs DM.

**Figure S3** Chemical structures of the compounds identified in GQD

P: Pueraria Lobatae Radix; S: Scutellariae Radix; C: Coptidis Rhizoma; G: Glycyrrhizae Radix et Rhizoma Praeparata cum Melle.

**Figure S4** Extracted ion chromatograms of 133 constituents from GQD.

P: Pueraria Lobatae Radix; S: Scutellariae Radix; C: Coptidis Rhizoma; G: Glycyrrhizae Radix et Rhizoma Praeparata cum Melle.

**Figure S5** PCA score plots of GQD and FGQD. A: negative ion; B: positive ion.

**Figure S6** Representative HPLC chromatograms of ten marker compounds at 254 nm and 276 nm.

P11: puerarin, P18: daidzin, P35: daidzein, C14: coptisine, C18: palmatine, C19: berberine, G3: liquiritin, G12: liquiritigenin, S19: baicalin, S37: baicalein.

Table S1 Calibration curves, LODs, LOQs, repeatability, accuracy and stability of the quantitative assays for 10 analytes in GQD

| Analytes | Linearity | | | LODs  (ng/mL) | LOQs  (ng/mL) | Precision (RSD%, n=6) | |  | Repeatability RSD (%) | |  | Stability (n=6) |  | Recovery (n=6) | |
| --- | --- | --- | --- | --- | --- | --- | --- | --- | --- | --- | --- | --- | --- | --- | --- |
|  | Range (μg/mL) | Calibration curve | r^2^ (n=6) |  |  | Inter-day | Intra-day |  | Mean concentration  (mg/g) | RSD (%) |  | RSD (%) |  | Average Recovery | RSD (%) |
| Puerarin | 17.70-247.80 | y=4×10^7^x+9074.7 | 0.9998 | 3.28 | 59.00 | 0.83 | 0.38 |  | 59.52 | 1.51 |  | 0.24 |  | 97.21 | 1.99 |
| Daidzin | 1.21-84.56 | y=4×10^7^x+10870 | 0.9997 | 60.40 | 120.80 | 0.53 | 0.36 |  | 2.16 | 0.60 |  | 0.68 |  | 93.08 | 1.89 |
| Daidzein | 1.82-50.99 | y=6×10^7^x-2586.2 | 0.9998 | 18.21 | 182.10 | 1.01 | 0.79 |  | 6.45 | 1.22 |  | 0.96 |  | 95.93 | 1.63 |
| Liquiritin | 1.00-10.02 | y=2×10^7^x+47.372 | 0.9987 | 50.10 | 100.20 | 0.93 | 0.87 |  | 0.74 | 1.02 |  | 1.59 |  | 97.31 | 1.48 |
| Liquiritigenin | 1.41-28.12 | y=4×10^7^x-7192.1 | 0.9996 | 70.30 | 140.60 | 0.88 | 1.90 |  | 1.98 | 0.83 |  | 1.90 |  | 97.81 | 1.69 |
| Coptisine | 10.55-49.21 | y=8×10^6^x+7385 | 0.9994 | 527.25 | 1054.50 | 0.96 | 0.45 |  | 3.63 | 1.85 |  | 1.00 |  | 95.02 | 1.98 |
| Berberine | 4.86-68.04 | y=1×10^7^x+3600.1 | 0.9995 | 243.00 | 486.00 | 1.32 | 0.73 |  | 9.67 | 1.62 |  | 0.72 |  | 96.16 | 2.08 |
| Palmatine | 2.04-32.64 | y=1×10^7^x+2018.6 | 0.9997 | 102.00 | 204.00 | 2.00 | 0.78 |  | 6.61 | 1.98 |  | 0.86 |  | 98.54 | 1.28 |
| Baicalin | 3.22-221.81 | y=2×10^7^x-24422 | 0.9997 | 32.19 | 321.92 | 0.67 | 0.40 |  | 24.16 | 0.68 |  | 0.43 |  | 95.62 | 2.55 |
| Baicalein | 1.50-104.65 | y=2×10^7^x-18868 | 0.9997 | 74.75 | 149.50 | 2.00 | 1.74 |  | 0.67 | 0.46 |  | 1.82 |  | 95.09 | 0.52 |

**
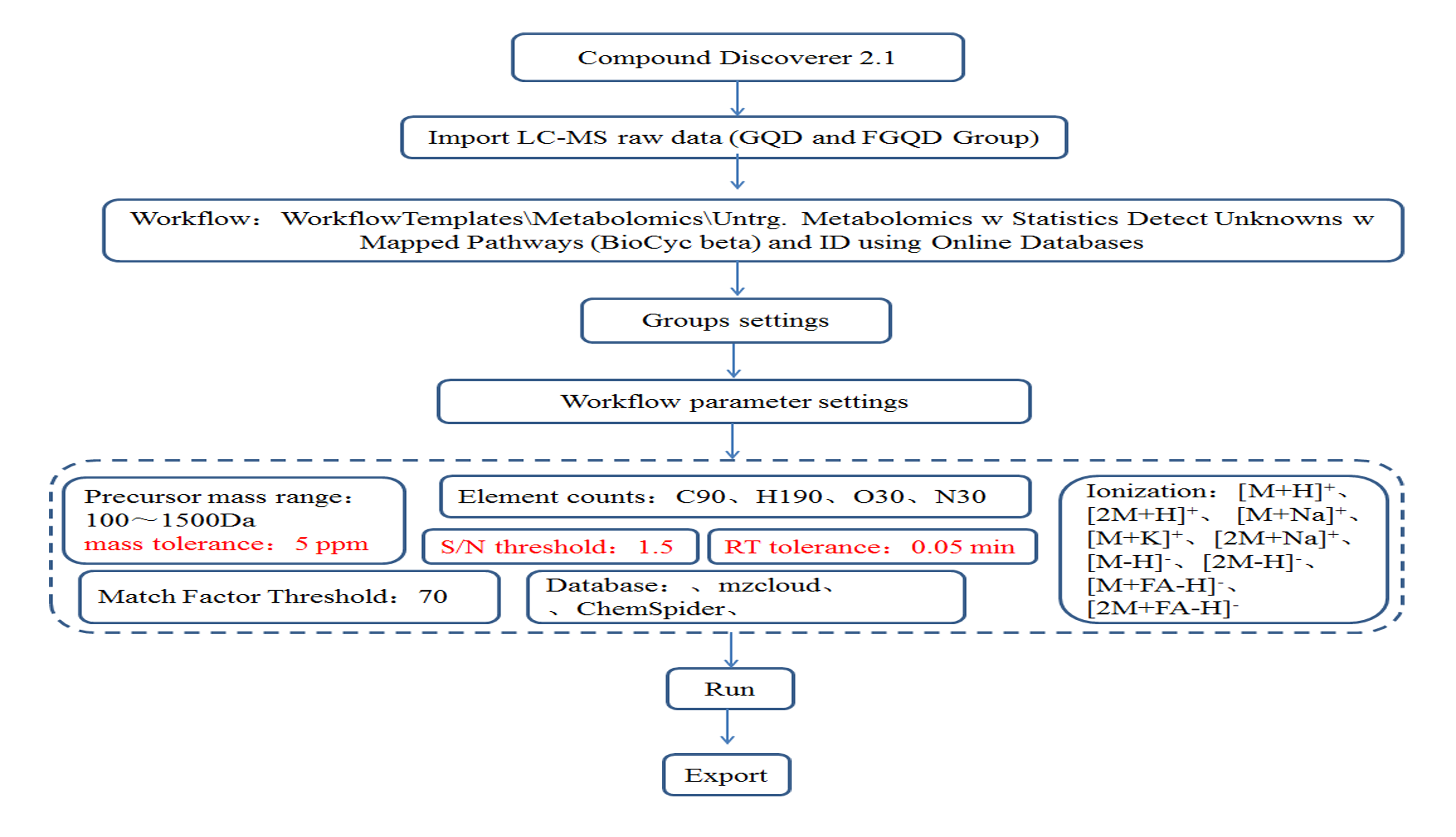
Figure S1**

**Figure S2**


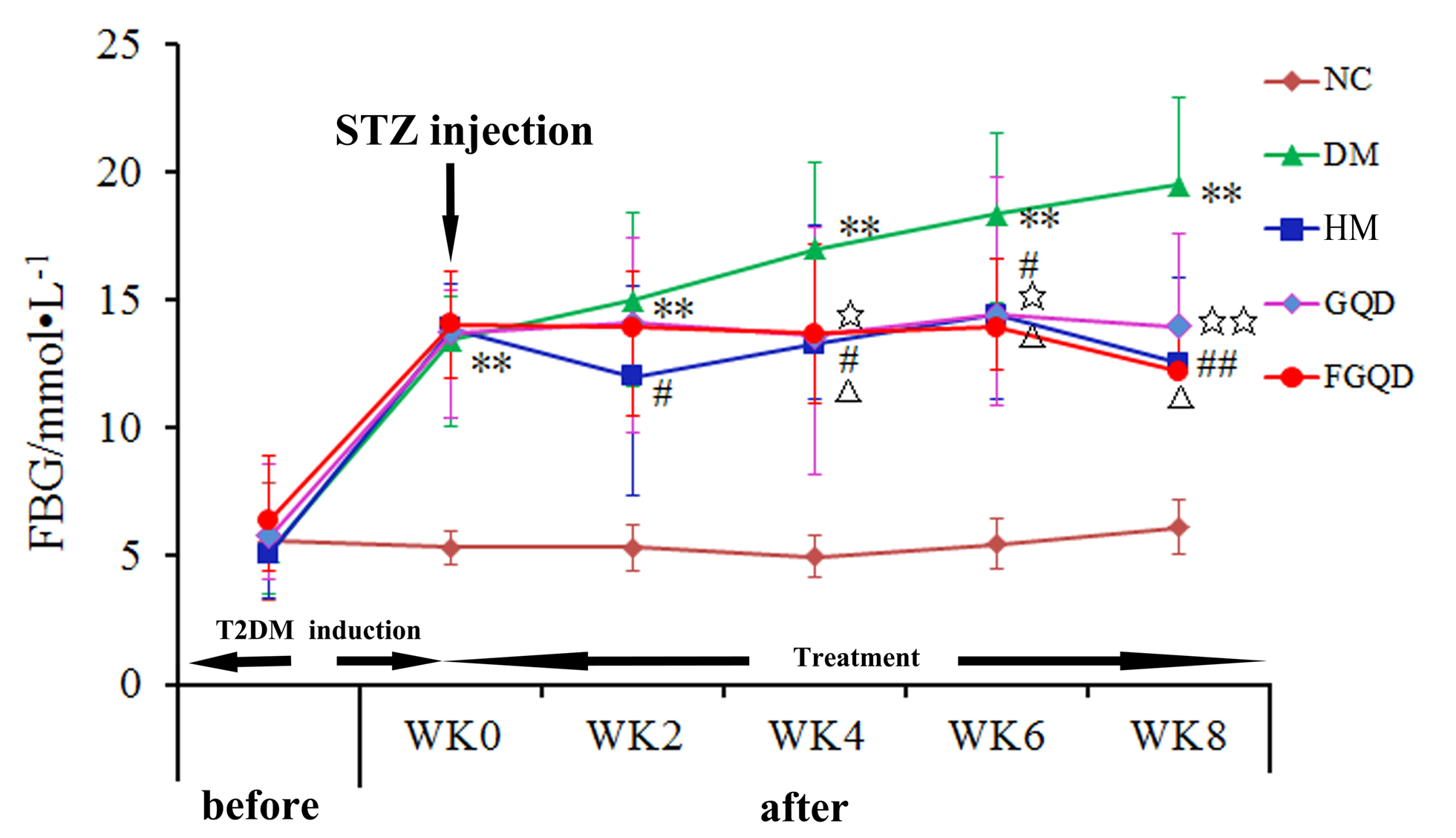


**Figure S3**

**
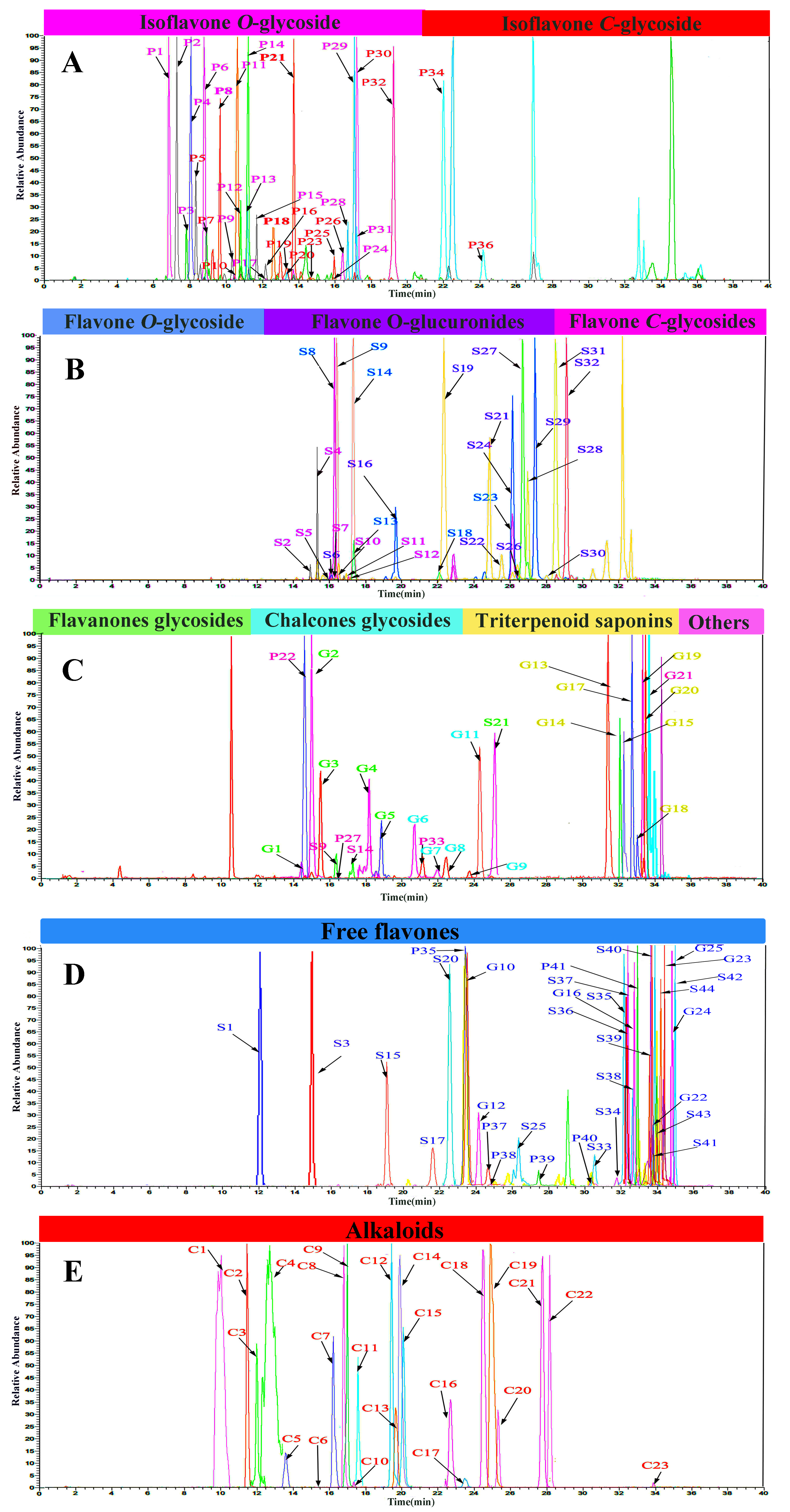
Figure S4**

**
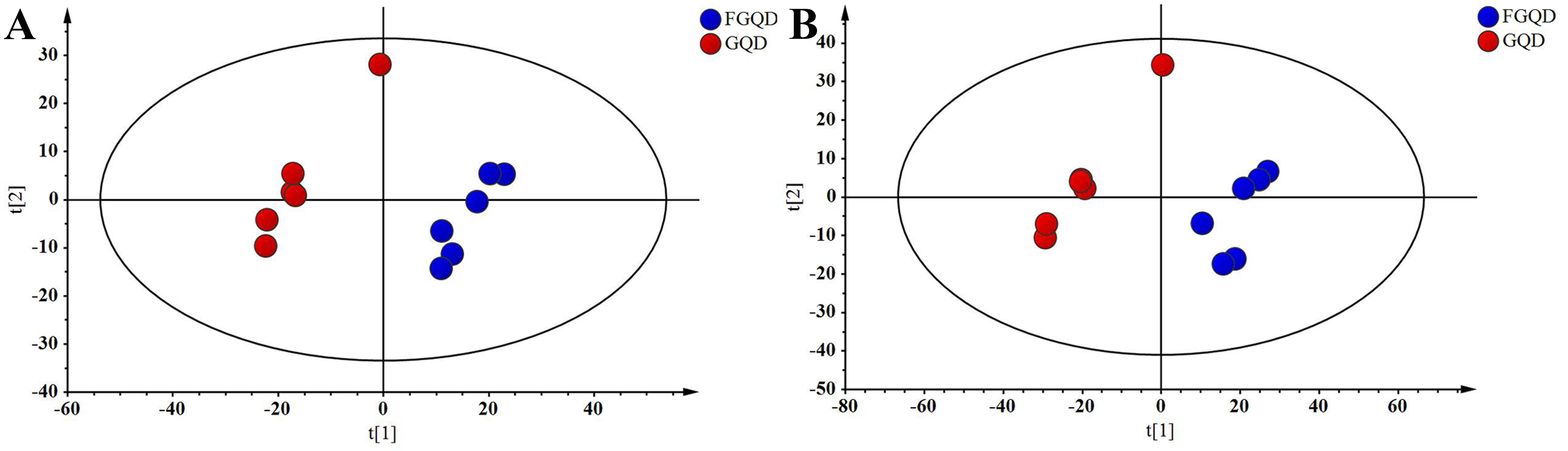
Figure S5**

**Figure S6**


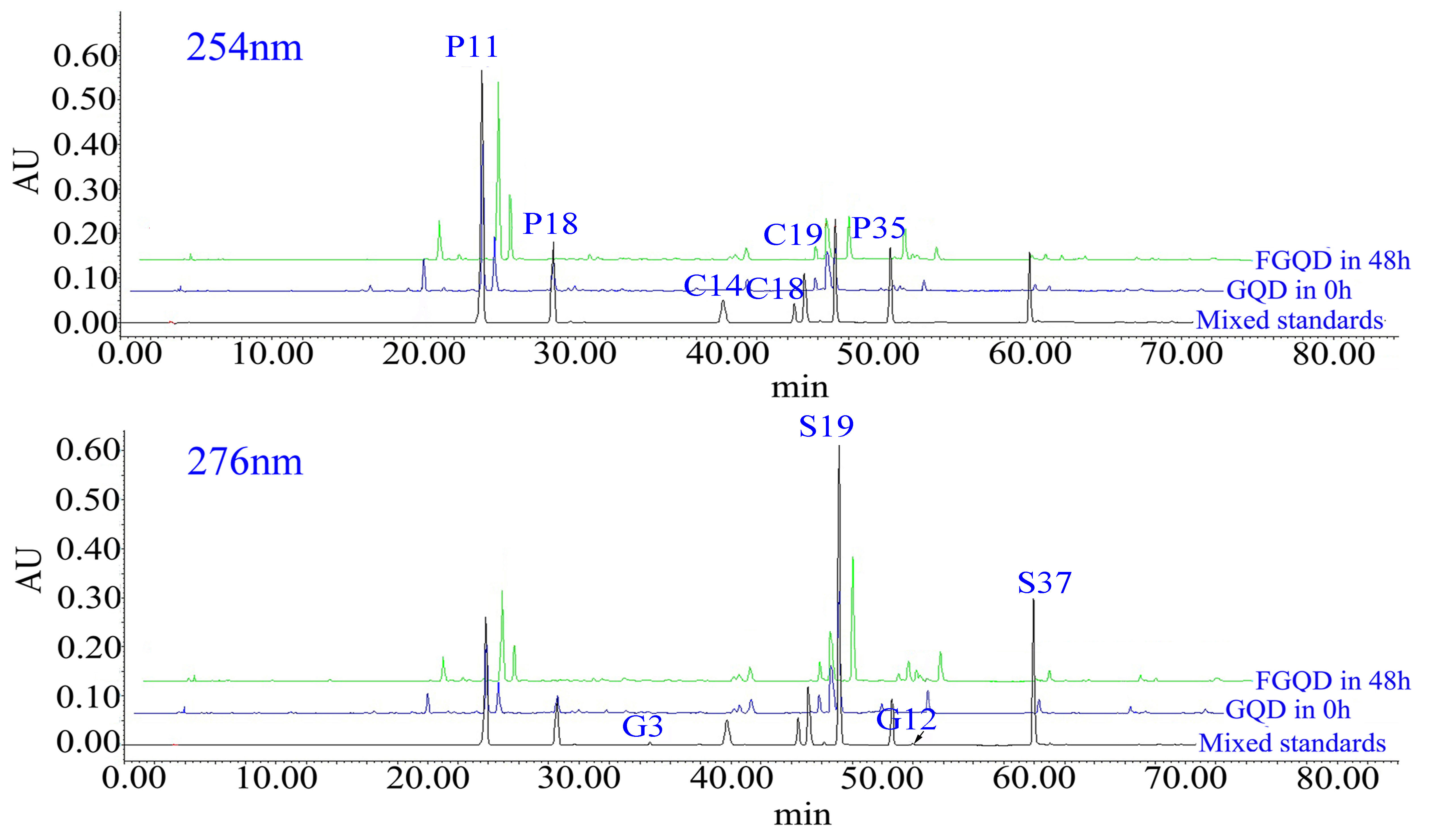

Supplement: Supplementary file 2 — Additional file 2: Table S1. Calibration curves, LODs, LOQs, repeatability, accuracy and stability of the quantitative assays for 10 analytes in GQD. Figure S1. Workflow of the untargeted metabolomic analysis. Figure S2 Effects of HM, GQD and FGQD on the FBG levels in T2DM rats. **p<0.01 DM vs NC; #p<0.05, ##p<0.01 HM vs DM; ☆p<0.05, ☆☆p <0.01 DM vs GQD; △p<0.05, △△p <0.05 FGQD vs DM. Figure S3. Chemical structures of the compounds identified in GQD. P: Pueraria Lobatae Radix; S: Scutellariae Radix; C: Coptidis Rhizoma; G: Glycyrrhizae Radix et Rhizoma Praeparata cum Melle. Figure S4. Extracted ion chromatograms of 133 constituents from GQD. P: Pueraria Lobatae Radix; S: Scutellariae Radix; C: Coptidis Rhizoma; G: Glycyrrhizae Radix et Rhizoma Praeparata cum Melle. Figure S5. PCA score plots of GQD and FGQD. A: negative ion; B: positive ion. Figure S6. Representative HPLC chromatograms of ten marker compounds at 254 nm and 276 nm. P11: puerarin, P18: daidzin, P35: daidzein, C14: coptisine, C18: palmatine, C19: berberine, G3: liquiritin, G12: liquiritigenin, S19: baicalin, S37: baicalein. [file 13020_2018_208_MOESM2_ESM.doc]
